# Reduced-dimensional perovskite photovoltaics with homogeneous energy landscape

**DOI:** 10.1038/s41467-020-15451-1

**Published:** 2020-04-03

**Authors:** Tingwei He, Saisai Li, Yuanzhi Jiang, Chaochao Qin, Minghuan Cui, Lu Qiao, Hongyu Xu, Jien Yang, Run Long, Huanhua Wang, Mingjian Yuan

**Affiliations:** 1 Key Laboratory of Advanced Energy Materials Chemistry (Ministry of Education), Renewable Energy Conversion and Storage Center (RECAST), College of Chemistry, Nankai University, 300071 Tianjin, People’s Republic of China; 2 Henan Key Laboratory of Infrared Materials and Spectrum Measures and Applications, Henan Normal University, 453007 Xinxiang, People’s Republic of China; 3 College of Chemistry, Key Laboratory of Theoretical and Computational Photochemistry of Ministry of Education, Beijing Normal University, 100875 Beijing, People’s Republic of China; 4 Institute of High Energy Physics, Chinese Academy of Science, 100049 Beijing, People’s Republic of China

**Keywords:** Chemistry, Energy science and technology, Materials science

## Abstract

Reduced-dimensional (quasi-2D) perovskite materials are widely applied for perovskite photovoltaics due to their remarkable environmental stability. However, their device performance still lags far behind traditional three dimensional perovskites, particularly high open circuit voltage (*V*_oc_) loss. Here, inhomogeneous energy landscape is pointed out to be the sole reason, which introduces extra energy loss, creates band tail states and inhibits minority carrier transport. We thus propose to form homogeneous energy landscape to overcome the problem. A synergistic approach is conceived, by taking advantage of material structure and crystallization kinetic engineering. Accordingly, with the help of density functional theory guided material design, (aminomethyl) piperidinium quasi-2D perovskites are selected. The lowest energy distribution and homogeneous energy landscape are achieved through carefully regulating their crystallization kinetics. We conclude that homogeneous energy landscape significantly reduces the Shockley-Read-Hall recombination and suppresses the quasi-Fermi level splitting, which is crucial to achieve high *V*_oc_.

## Abstract (web summary)

Quasi-2D halide perovskites are good candidates for efficient and stable solar cells but they suffer from high open-circuit voltage (*V*_oc_) deficit. Here He et al. show that homogeneous energy landscape of the charge transport levels is important to suppressing the recombination and increasing *V*_oc_.

## Introduction

The certified power conversion efficiency (PCE) of three-dimensional (3D) perovskite solar cells (PSCs) has reached 25.2%^1,2^. However, the device stability is still undesirable, which severely hinders their commercialization process^3,4^. To date, reduced-dimensional (quasi-2D) perovskites are believed to be the most successful attempt to address the instability problems^5,6^. Quasi-2D perovskite is generated by slicing 3D perovskite along one of its crystallographic plane, and subsequently incorporating bulky organic cations to separate the inorganic slabs^7^. The resulting derivatives exhibit greatly improved stability, especially the low ‘quantum-well’ thickness (<*n*> value) species^8,9^. However, PCE for these quasi-2D photovoltaics still lags far behind their 3D counterpart. In particular, the devices display low open-circuit voltage (*V*_oc_), indicating the existence of non-negligible nonradiative recombination.

Further performance improvement solely relies on deeper understanding of the device operation mechanism. However, carrier transport in quasi-2D films is complicated and still quite elusive to date. As far as we know, multiple <*n*> value species are created during the crystallization. Accordingly, efficient cascade energy transfer takes place among different <*n*> value species, and the carriers would eventually concentrate to the lowest bandgap species^10^. Great success has accomplished in perovskite light-emitting diodes (PeLEDs) by taking advantage of this phenomenon^11^. Nevertheless, photovoltaics rely on completely opposite operation mechanism compared with PeLEDs, which requires carriers to get separated rather than get recombined. Therefore, we point out that the aforementioned cascade energy transfer that is caused by the inhomogeneous energy landscape will degenerate photovoltaic performance due to the following reasons.

First, energy transfer dynamics is demonstrated to dominate the carrier transport in quasi-2D films, because the cascade energy transfer typically happens at a very early stage after photoexcitation. From the viewpoint of carrier dynamics, carrier transit time across the *p*-*n* junction is much longer than the energy transfer timescale, when considering the carrier mobility and film thickness^12^. This implies that the photocarriers generating in the high bandgap species would thermally relax to the band edge of the smallest bandgap species at first, and then get separated under the built-in electric field. This cooling process inevitably results in extra energy loss, and the overall device’s *V*_oc_ is thus limited by the smallest bandgap species^13^. Second, severe energy disorder exists in the quasi-2D perovskite films, which is mainly induced by the inhomogeneous energy landscape^14^. High degree of energy disorder widens the distribution of electronic states and creates band tail states, which have been proved to lead to notable *V*_oc_ loss in both organic and quantum dot photovoltaics^15,16^. Hence, it is foreseen that quasi-2D perovskite photovoltaics should suffer from the energy disorder as well. In summary, the inhomogeneous energy landscape deteriorates the device performance by introducing extra energy loss, creating band tail states, and inhibiting minority carrier transport. Therefore, quasi-2D perovskite film with homogeneous energy landscape is proposed here to address the problems. However, the approach is still highly challenging and rarely explored yet.

Fortunately, significant advances have been made toward deeply understanding the quasi-2D film’s formation kinetics and the resulting band structure. Proppe et al.^17^ first reported a quasi-2D perovskite film whose bandgaps concentrated at 1.65 eV, through judiciously selecting the allylammonium ligand. Zhou et al.^18^ also demonstrated that the <*n*> value distribution could be controlled by introducing methylammonium chloride additive. Subsequently, Shao et al.^19^ successfully tuned the energy landscape of perovskite films via a vacuum-assisted method. Reduced low <*n*> value components favorably promoted the carrier transport and effectively suppressed the charge recombination. These films featuring high <*n*> value phases achieved improved performance. Nevertheless, to some extent, they sacrificed the natural stability advantages of quasi-2D perovskites. Therefore, achieving homogeneous energy landscape in low <*n*> value regions is necessary to ensure both performance and stability of the PSCs.

The knowledge thus leverages us an opportunity to realize homogeneous energy landscape via material design and crystallization dynamics engineering. According to previous report, the polydispersity of <*n*> values is highly dependent on the spatial distribution of organic cations in precursor solution^14^. In principle, narrow <*n*> value-distributed quasi-2D films are formed, once the organic cations become homogeneously distributed. Moreover, it is reasonable to minimize the band offsets between adjacent <*n*> value species by judiciously selecting the material structure. The reduced band offsets thereby flatten the overall band structure, and help to create the homogeneous energy landscape.

Based on the above inspiration, we thus conceive a synergistic approach to realize homogeneous energy landscape by taking advantage of both material structure and crystallization kinetic regulation. With the help of the density functional theory (DFT) simulation, a *meta*-(aminomethyl) piperidinium (MAMP) quasi-2D perovskite film is fabricated, which possesses an overall flatter band structure. Furthermore, MAMP film with a homogeneous energy landscape is realized by carefully regulating the crystallization kinetics. The energy dispersion of the corresponding film is much smaller than the control, *n*-butylammonium (BA) film. Electrical characterizations confirm that the homogeneous energy landscape decreases the defect density and reduces the film’s energy disorder. We then correlate the relationship between homogeneous energy landscape and device performance. The physical origin of *V*_loss_ has been thoroughly investigated by various electrical analyses and solar cell capacitance simulator (SCAPS) simulation. Minimal *V*_loss_ was achieved owing to the reduced nonradiative recombination and energy disorder, which is induced by the homogeneous energy landscape. The device exhibits an excellent PCE of 16.53%, accompanied by a high *V*_oc_ of 1.21 V. In addition, the device exhibits significantly improved stability compared with traditional hot-casting films due to the more ideal vertical phase alignment, which retains almost 90% of the initial PCE after 1000 h of storage.

## Results

### DFT simulation-guided material design

**Fig. 1:**
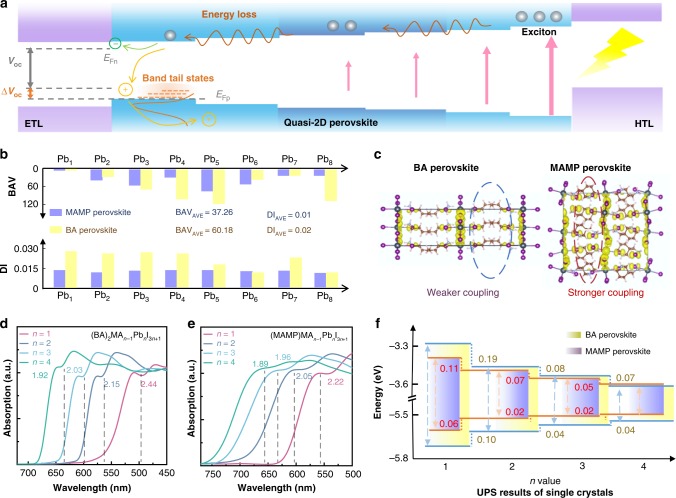
Device operation mechanism and DFT simulation. **a** Operation mechanism of quasi-2D perovskite photovoltaics and the correlation between band structure, energy disorder, and *V*_oc_ of the device. **b** [PbI_6_]^4−^ octahedron distortion for (BA)_2_(MA)_3_Pb_4_I_13_ and (MAMP)(MA)_3_Pb_4_I_13_, and the resulting BAV and DI. **c** Spinor density for (BA)_2_PbI_4_ and (MAMP)PbI_4_ perovskites illustrating interlayer I–I interactions. Absorption spectra for **d** (BA)_2_MA_*n*−1_Pb_*n*_I_3*n*+1_ and **e** (MAMP)MA_*n*−1_Pb_*n*_I_3*n*+1_ (*n* = 1, 2, 3, 4) single crystals. **f** CBM and VBM positions of (BA)_2_(MA)_*n*−1_Pb_*n*_I_3*n*+1_ and (MAMP)(MA)_*n*−1_Pb_*n*_I_3*n*+1_ perovskite single crystals (*n* = 1, 2, 3, 4) measured by UPS.

Flattening the overall band structure of a quasi-2D film is equal to reducing the band offsets between the adjacent quasi-2D species (Fig. 1a). Accordingly, the goal can be indirectly realized by minimizing the individual quasi-2D perovskites’ bandgap. Organic cations can affect the quasi-2D perovskites’ lattice structure via versatile hydrogen bond types. The discrepancy makes huge impact on the resulting energy level through the degree of octahedral tilting and interlayer electronic coupling strength. Accordingly, interlayer electronic coupling participates in antibonding interactions via van der Waals halide–halide interactions. Short halide–halide distance affords strong antibonding interactions, which push up the valence band maximum (VBM) in principle^20^. Meanwhile, octahedral tilting affords new hybrid orbitals by mixing *s* and *p* orbitals of halide. The hybridization determines the conduction band minimum (CBM) antibonding between *p* orbitals of metal and *s*/*p* hybrid orbitals of halide. Small octahedral tilting pushes up the VBM and pushes down the CBM, resulting in the reduced bandgap^21,22^.

Accordingly, minimizing the octahedral tilting and reducing the interlayer distance are the keys to flatten the band structure, which requires us to judiciously examine the organic cation and the resulting lattice geometry. We noticed that a series of Dion-Jacobson phase (DJ) quasi-2D perovskites that were first reported by Kanatzidis and colleagues^20^ might potentially meet the requirements^20^. The DJ perovskites were characterized with divalent interlayer spacers. The immobile divalent cations pushed the inorganic slabs close to each other. Accordingly, MAMP quasi-2D perovskites exhibited the shortest interlayer halide–halide distance (about 4.0 Å) among all the quasi-2D perovskites reported to date (Supplementary Fig. 1)^23,24^. In addition, negligible octahedral tilting was found in MAMP perovskites, since the axial Pb–I–Pb angles are close to 180° (Supplementary Fig. 2). In principle, short halide–halide distance and negligible octahedral tilting should decrease the bandgap, which subsequently reduced the band offsets and enabled the films to possess a relatively flatter overall band structure.

DFT simulation was used to simulate the band structure for perovskites (Supplementary Note 1). Detailed [PbI_6_]^4−^ octahedron distortion was calculated through the bond angle variance (BAV) and distortion index (DI) (Fig. 1b)^25,26^. MAMP perovskite exhibited a smaller average BAV and DI than BA perovskite, indicating less octahedral tilting. In addition, we simulated the spinor density to illustrate the interlayer coupling strength of I–I (Fig. 1c). As expected, MAMP perovskite reflected stronger antibonding interactions compared with BA perovskite. As a result, MAMP perovskites exhibited a smaller bandgap compared with regular BA perovskites (Supplementary Figs. 3 and 4).

High-quality (MAMP)MA_*n*−1_Pb_*n*_I_3*n*+1_ and (BA)_2_MA_*n*−1_Pb_*n*_I_3*n*+1_ (*n* = 1, 2, 3, 4) single crystals (SCs) (Supplementary Figs. 7–9) were obtained by an improved solvent growth method. CBM and VBM positions for these SCs were measurement through their absorption spectra (Fig. 1d, e) and ultraviolet photoelectron spectrometer (UPS) (Fig. 1f, Supplementary Figs. 5 and 6, and Supplementary Tables 1 and 2). As shown, the measured bandgap for MAMP SCs’ each *n* value was greatly smaller than BA ones. Thereby, VBM and CBM band offsets between adjacent *n* values reduced in MAMP SCs. Consequently, the bandgap energy dispersion from *n* = 1 to *n* = 4 species was calculated to be 0.33 eV for MAMP perovskite, which was much lower than BA case (0.52 eV).

### Crystallization kinetics regulation

**Fig. 2:**
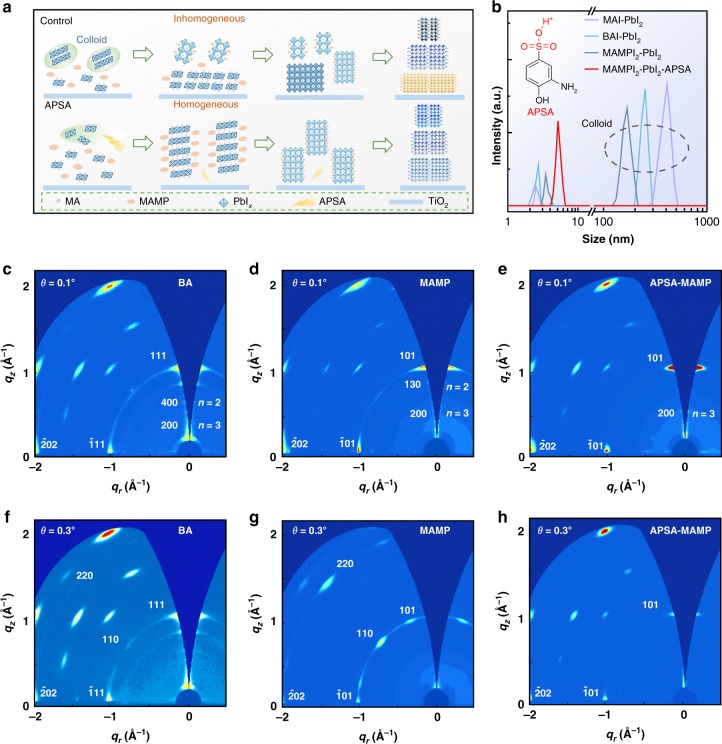
Crystallization kinetics regulation. **a** Schematic models indicating crystallization process for both control and APSA-treated quasi-2D perovskite films. **b** DLS data to evaluate the colloid size and distribution in precursor solutions. Inset: molecular structure of APSA. GIWAXS characterizations for (BA)_2_(MA)_3_Pb_4_I_13_, pristine, and APSA-treated (MAMP)MA_3_Pb_4_I_13_ perovskite films, **c**–**e** with grazing-incidence angle of 0.1° and **f**–**h** with grazing-incidence angle of 0.3°, respectively.

Corresponding quasi-2D films were fabricated by single-step spin-coating process with the aid of antisolvents. The quasi-2D perovskite films’ formation kinetics need to be well controlled to achieve narrow <*n*> value distribution. Previous research demonstrated that colloidal precursor sol-gel, MA^+^/PbI${}_{x^{-}}$ complexes, formed spontaneously in the precursor solution^27^. The colloids provided nucleation sites for the subsequent crystallization (Fig. 2a), which accelerated the crystallization speed locally. Lower <*n*> value quasi-2D perovskites crystallized with high priority, since they possess lower crystallization energy barrier compared with higher <*n*> value analogs. As a result, local organic cation shortage appeared due to the intense consummation of organic cations. Accordingly, higher <*n*> value quasi-2D or quasi-3D perovskites precipitated in sequence, leading to a broad <*n*> value distribution.

Apparently, eliminating the complex colloids is essential to avoid the broad <*n*> value distribution. Previous research has demonstrated the manipulation of the colloidal concentration by modulating the solution pH^28^. In particular, acidic additives were shown to be able to dissolve the pre-formed colloids. Accordingly, here we reported introducing 2-aminophenol-4-sulfonic acid (APSA) to suppress the colloids’ formation (Supplementary Fig. 11). Dynamic light scattering (DLS) was used to monitor the colloids’ size and concentration. As shown in Fig. 2b, the colloids with the average size around 400, 250, and 180 nm were observed for MAPbI_3_, BA, and pristine MAMP precursor solution, respectively. In contrast, no colloid was found in MAMP precursors treated with APSA, indicating that colloids have been suppressed. We also investigated the resulting films’ crystallization rate by recording the evolution of absorption spectra (Supplementary Fig. 12). As shown, the crystallization time for APSA-treated MAMP film was much longer than the pristine MAMP film, illustrating that total nucleation sites decreased. The phenomenon was in coherence with the DLS interpretation. Moreover, APSA-treated MAMP film exhibited the least high <*n*> value phases by comparing their absorption spectra (Supplementary Figs. 13–15).

We then investigated the quasi-2D perovskite’ crystal orientation and phase composition by grazing-incidence wide-angle X-ray scattering (GIWAXS). All the films displayed the sharp and discrete Bragg spots (Fig. 2c–e), indicating that the crystals were highly oriented and packed. The discrete Bragg spots were assigned to (1 1 1), (1 0 1), and (2 0 2) planes through indexing the Bragg peaks. The dominant in-plane stacking of the (1 0 1), (2 0 2) facets points to the (*h* *0* *l*) facets that were oriented parallel to the substrate, confirming the inorganic slabs perpendicular to the substrate. As a result, highly vertically oriented quasi-2D perovskite films finally formed. Moreover, the GIWAXS patterns indicated the existence of the <*n*> = 2 phase in BA and pristine MAMP films, which was not observed in APSA-treated MAMP film. We further investigated whether vertical <*n*> value distribution was increased by the grazing-incidence angles (Fig. 2f–h). Debye-Scherrer rings, (1 1 0) and (2 2 0) planes, belonging to high <*n*> values or 3D perovskites, were observed at the bottom of BA and pristine MAMP films with a larger grazing-incidence angle. However, APSA-treated MAMP film exhibited almost identical Bragg spots, which support that the narrowest <*n*> value distribution existed in the film.

### Carrier dynamics in quasi-2D perovskite film

**Fig. 3:**
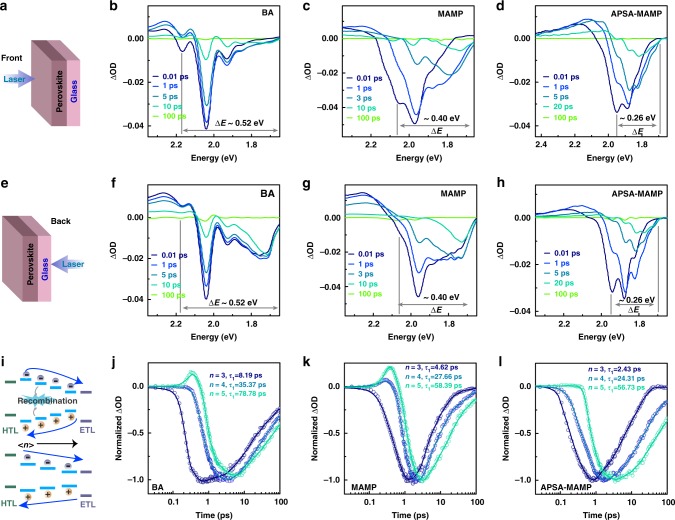
Carrier dynamics of quasi-2D perovskite films. Schematic of quasi-2D perovskite films excited from **a** the front side (air side) and **e** the back side (glass side). The corresponding TA spectra at different delay times of **b**, **f** (BA)_2_(MA)_3_Pb_4_I_13_, **c**, **g** pristine, and **d**, **h** APSA-treated (MAMP)MA_3_Pb_4_I_13_ films, respectively. **i** Schematic model showing internal charge transfer from low <*n*> value to high <*n*> value phases. **j**–**l** TA kinetics extracted from different GSB (*n* = 3, 4, 5) at front-side excitation for different films.

Ultrafast transient absorption (TA) spectroscopy technique was utilized to investigate carrier dynamics in quasi-2D perovskite films. The films were initially pumped from the front side (air side), and Fig. 3a–d showed their ground-state bleaching (GSB) signals. As expected, some obvious GSB peaks were displayed in each TA spectra, indicating that mixed <*n*> value species coexisted in films. However, the energy distribution, Δ*E*, for a pristine MAMP film was calculated to be about 0.40 eV, which was smaller than the control BA one (about 0.52 eV). The reason for this was due to the reduced band offsets in pristine MAMP film, and the phenomenon was consistent with SC characterization. Notably, as shown in Fig. 3d, <*n*> = 2 GSB peak disappeared in APSA-treated MAMP film, which was in agreement with GIWAXS data. Furthermore, GSB signals ascribed to the larger <*n*> value species also significantly diminished after APSA treatment. In short, TA data confirmed that the narrowest <*n*> value-distributed film was realized after APSA treatment. Δ*E* for APSA-treated MAMP film reduced to about 0.26 eV, owing to the synergistic effect of the band structure and crystallization dynamics management. It is worth mentioning that Δ*E* represents the narrowest energy distribution for low *<n*> value quasi-2D perovskite films reported to date.

**Fig. 4:**
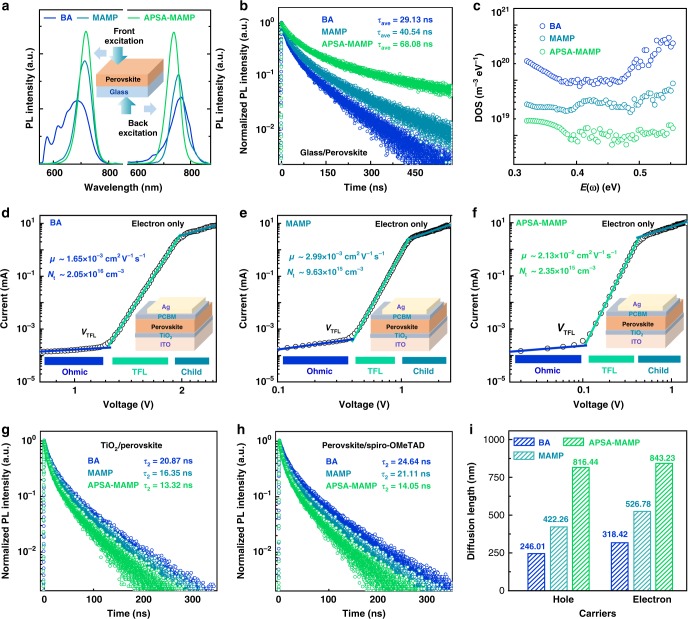
Optoelectronic properties of quasi-2D perovskite films. **a** Steady-state PL spectra of quasi-2D perovskite films excited from the front and back sides (as illustrated in the inset). **b** TRPL decay traces for three films based on glass substrate. **c** TAS analysis of three quasi-2D perovskite films. SCLC curves of **d** (BA)_2_(MA)_3_Pb_4_I_13_, **e** pristine, and **f** APSA-treated (MAMP)MA_3_Pb_4_I_13_ films, respectively. TRPL decay traces for three perovskites films covered with **g** TiO_2_ and **h** Spiro-OMeTAD layers. **i** Carrier diffusion length for both electron and hole was calculated from the optical method.

The films’ vertical phase distribution was examined via TA and photoluminescence (PL) spectra. As shown in Fig. 3e–h, pumping direction had an obvious effect on the GSB peak position. When the films were pumped from the glass side, the GSB peak intensity for higher <*n*> value species greatly enhanced compared with air-side pumping. The differences became more obvious in PL spectra. As shown in Fig. 4a, luminescence peak featuring of low <*n*> values governed the PL spectra in air-side excitation case. On the contrary, high <*n*> value PL peaks dominated when excited from the glass side. Accordingly, both TA and PL data implied that low <*n*> value phases prefer to stay close to the perovskite film surface, while the high <*n*> value species tend to stay at the bottom. Particularly, the vertical phase distribution of our films was totally reverse compared with the traditional hot-casting films, in which the high <*n*> value phases tend to stay on the top. Apparently, our films should display improved film stability compared with the traditional films. In addition, our films should be more suitable for the conventional device architecture, on account of the vertical energy-level alignment^18^. In short, graded MAMP quasi-2D perovskite films with homogeneous energy landscape have been achieved here.

Detailed carrier dynamics was analyzed by extracting decay kinetics from TA spectra. As shown in Fig. 3i–l, spontaneous electron transfer from low <*n*> value species to high <*n*> value species was observed in all films. Figure 3l illustrates the dynamic evolution of APSA-treated MAMP film. The <*n*> = 3 bleaching appeared at 0.1 ps, then an ultrafast decay was observed. The low <*n*> bleaching recovery kinetics was accompanied with the buildup of the high <*n*> bleaching, and the formation of the <*n*> = 5 bleaching was delayed to 0.4 ps. Moreover, BA and pristine MAMP films displayed slow charge transfer dynamics, which would lead to charge accumulation in low <*n*> value species. It thus increased the possibility of exciton annihilation and minority carrier recombination. As expected, APSA-treated MAMP film displayed the fastest decay time in the low <*n*> value domain. The same result could be obtained by its steady-state PL spectra (Fig. 4a). A strikingly strong and sharp PL peak appeared in APSA-treated MAMP film. The relative small band offsets between adjacent <*n*> value species certainly reduced the energy barrier and facilitated the carrier migration. The ultrafast charge transfer process then suppressed the carrier recombination.

### Optoelectronic property characterization

Carrier dynamics analysis indicated that homogeneous energy landscape should induce less defect density, increased minority carrier mobility, and elongated diffusion length. Detailed optical and electrical characterizations have been carried out to confirm the speculation. We first extracted the films’ carrier lifetime through time-resolved PL (TRPL) measurement. As shown in Fig. 4b, the average decay lifetime (*τ*_ave_) for APSA-treated MAMP film was longer than the other two films, suggesting reduced nonradiative recombination. Efficient minority carrier transport is very essential in photovoltaic operation, which plays a key role in determining the quasi-2D device performance. Accordingly, we extracted electron mobility for these films through the space-charge-limited current (SCLC) technique. As shown in Fig. 4d–f, the electron mobility for BA film was determined to be 1.65 × 10^−3^ cm^2^ V^−1^ s^−1^, which was consistent with the previous report. In contrast, APSA-treated MAMP film exhibited one order of magnitude higher electron mobility, 2.13 × 10^−2^ cm^2^ V^−1^ s^−1^, indicating more efficient minority carrier transport realized in this homogeneous energy landscape.

The elongated carrier lifetime and enhanced minority carrier mobility indicated reduced defect density. Through SCLC, we investigated the defect density to understand its physical origins. As expected, the defect density for APSA-treated MAMP film was found to be more than one order of magnitude lower than BA or pristine MAMP films. It is controversial whether the SCLC technique can accurately evaluate the defect density for perovskites, considering the notorious ‘ion migration.’ We thereby measured the defect density again through thermal admittance spectroscopy (TAS), to characterize shallow and deep defects of quasi-2D perovskite films^29^. As shown in Fig. 4c, APSA-treated MAMP film again exhibited the lowest defect density of states (*t*DOS) over the whole trap depth. The inherent trap density of MAMP perovskite crystal was lesser than that of BA one (Supplementary Fig. 10). Furthermore, sulfonic acid passivation and homogeneous energy landscape contributed to reducing defect states^30,31^. As a result, APSA-treated MAMP film exhibited the lowest trap-assisted recombination, as shown in density-dependent TRPL spectra (Supplementary Fig. 16).

We evaluated the diffusion length (*L*_D_) via a well-established optical method. A hole or electron quencher was placed on the top of perovskite films, and a fast PL quenching was found on APSA-treated MAMP film (Fig. 4g, h and Supplementary Fig. 17). The corresponding hole (*L*_D–h_) or electron (*L*_D–e_) diffusion length could be extracted by comparing their bimolecular recombination lifetime with and without the quencher^32^. The approximate value of *L*_D_ was estimated by using the following equation:

1 $$L_{\mathrm{D}} \approx \frac{{2d}}{\pi }\sqrt {2\left( {\frac{{\tau _2}}{{\tau _{2,{\mathrm{quench}}}}} - 1} \right)},$$

where *d* is the film thickness, *τ*_2,quench_ and *τ*_2_ represent the bimolecular recombination lifetime with and without the quencher. As shown in Fig. 4i, the *L*_D–h_ and *L*_D–e_ for the control BA film were calculated to be about 200 and 300 nm, which was consistent with previous results^17^. Accordingly, APSA-treated MAMP film exhibited almost three times elongated diffusion length for both hole and electron.

The overall optical and electrical characterizations proved that significant improvement on semiconducting properties has reached by forming a homogeneous energy landscape. The outstanding semiconducting property of materials motivated us to further investigate the photovoltaic performance of devices, and deeply understand the physical origin at the device level. As aforementioned, our films’ vertical phase distribution was totally reverse to the traditional film. The resulting graded band structure indicated that conventional device architecture is more suitable for charge transport and collection.

### Relationship between energy disorder and *V*_oc_ loss

**Fig. 5:**
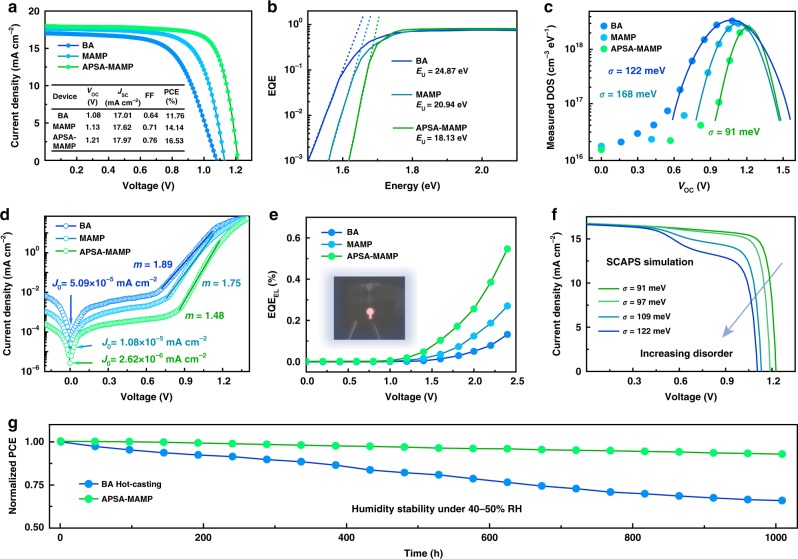
Photovoltaic performance and *V*_oc_ loss mechanism investigation. **a** *J–V* curves of (BA)_2_(MA)_3_Pb_4_I_13_, pristine, and APSA-treated (MAMP)MA_3_Pb_4_I_13_ devices. **b** *E*_U_ extracted by band edge fitting of the exponentially plotted EQE spectra. **c** DOS for different perovskites extracted from the impedance spectra. **d** Dark *J–V* curves for different perovskite devices. **e** EQE_EL_ spectra for three different perovskite devices. **f** SCAPS simulation of (MAMP)MA_3_Pb_4_I_13_ photovoltaics with experimental parameters extracted from different degrees of disorder films. **g** PCE evolution curves for unencapsulated APSA-treated (MAMP)MA_3_Pb_4_I_13_ devices, and the (BA)_2_MA_3_Pb_4_I_13_ devices fabricated by traditional hot-casting method, under humidity of 40–50% RH (relative humidity).

Planar photovoltaic devices with conventional architecture of ITO/TiO_2_/perovskite/Spiro-OMeTAD/Au were fabricated (Supplementary Fig. 18). The resulting device performance is shown in Fig. 5a. As shown, BA device delivered a PCE of 11.76%, with a *V*_oc_ of 1.08 V, and fill factor (FF) of 64%. The value well aligned with the reported PCE for low <*n*> value quasi-2D photovoltaics^8^. As revealed, the PCE mainly was limited by the relatively low FF and *V*_oc_, indicating inefficient carrier transport and collection. In particular, the *V*_oc_ loss determined as high as 0.55 V, in consideration of the bandgap of 1.63 V for <*n*> = 4 BA perovskite. Pristine MAMP perovskite possessed a relatively flatter band structure and exhibited a PCE of 14.14%, with slightly improved FF and reduced *V*_oc_ loss. Notably, a stable and hysteresis-free 16.53% PCE of APSA-treated MAMP perovskites was obtained (Supplementary Figs. 19a and 20–22 and Supplementary Table 3). It was encouraging that high *V*_oc_ (1.21 V) was obtained, indicating a very low *V*_oc_ loss (0.47 V) (Supplementary Fig. 23). From the *V*_oc_ evolution trend, it is obvious that flattening the overall band structure was beneficial in reducing *V*_oc_ loss.

The low *V*_oc_ loss motivated us to investigate its physical origin. As aforementioned, quasi-2D perovskite film is a mixed film with anisotropic transport, which severely suffers from energy disorder. The origin of the disorder can be classified as band structure, charge transport, and phase distribution disorder. It is a well-known energy disorder that introduces band tail states in the bandgap. The nonequilibrium photocarriers thus tend to relax down to these electronic states, thereby affecting the quasi-Fermi level splitting. Band tail states filled with carriers represent the effective bandgap (${E}_{{\mathrm{g}}}^{{\prime}}$) that determines the maximum attainable *V*_oc_ of quasi-2D perovskite device^16^. In principle, achieving low- energy disorder films with reduced band tail states is very critical, in order to minimize the *V*_oc_ deficit.

To evaluate the energy disorder in these films, we extracted the Urbach tail (*E*_U_). *E*_U_ was quantified via the density of localized band tail states; a higher *E*_U_ was indicative of more disorder^33^. *E*_U_ was quantified from the slope of the exponential external quantum efficiency (EQE) plots (Supplementary Fig. 24), which is proportional to exp[(*E* *−* *E*_g_)/*E*_U_]. As shown in Fig. 5b, the sharpest band tail was observed for APSA-treated MAMP perovskite device, which yields the lowest *E*_U_ of about 18.13 meV. It was worth mentioning that the extracted *E*_U_ was comparable to the values reported in 3D perovskite, since a room temperature Urbach tail of 13–15 meV was typically observed in high-quality 3D perovskite films.

Energy disorder at the device level could be directly probed by measuring the distribution of the electronic DOS. The distribution of DOS is expressed in the following equation:^34^

2 $$g\left( {E - E_{\mathrm{L}}} \right) = \frac{N}{{\sqrt {2\pi } \sigma }}{\mathrm{exp}}\left[ { - \frac{{\left( {E_{\mathrm{L}} - E} \right)^2}}{{2\sigma ^2}}} \right],$$

where *σ* is the disorder parameter; *N* and *E*_L_ represent the total density per unit volume and the energy center of the DOS, respectively. As we know, chemical capacitance (*C*_µ_) reflects the capability of accepting or releasing additional carriers for photovoltaics. The change in the capability shifts the quasi-Fermi level when it moves across the electronic states^35^. By fitting the data (Supplementary Fig. 25), APSA-treated MAMP perovskite exhibited the lowest *σ*, 91 meV (Fig. 5c). The variation trend for *σ* was consistent with the Urbach tail measurement, indicating that the homogeneous energy landscape did decrease the corresponding film’s energy disorder.

### *V*_oc_ loss mechanism at the device level

Besides, with the effective bandgap, the *V*_oc_ loss induced by nanoradiative recombination is another source to affect the attainable *V*_oc_. We then investigated the *V*_oc_ loss mechanism using classical semiconductor theory, to uncover the recombination pathway. *V*_oc_ can be expressed in this well-known equation:

3 $$eV_{{\mathrm{oc}}} = E_{\mathrm{g}}^{\prime} - k_{\mathrm{B}}T{\mathrm{ln}}\left( {\frac{{\beta N_{\mathrm{c}}N_{\mathrm{v}}}}{G}} \right),$$

where ${E}_{\mathrm{g}}^{\prime}$ represents the effective bandgap; *N*_c_ and *N*_v_ represent the effective DOS in conduction and valence bands; *k*_B_ is the Boltzmann constant; *G* is the carrier generation rate; *β* is the radiative recombination rate constant. The well-known diode ideality factor, *m*, is used to describe the dominating recombination mechanism. At the device level, the carrier generation rate *G* is the sum of thermal-generated portion *G*_t_ and optical portion *G*_opt_. The *G*_t_ is equal to the thermal recombination rate in the dark as

4 $$G_{\mathrm{t}} = \beta N_{\mathrm{c}}N_{\mathrm{v}}{\mathrm{exp}}\left( { - \frac{{E_{\mathrm{g}}}}{{k_{\mathrm{B}}T}}} \right).$$

The carrier generation rate can be experimentally measured from the reverse-bias saturation dark current density (*J*_0_) and reverse-bias saturation photocurrent density (*J*_sc_). We then can rewrite the equation as

5 $$V_{{\mathrm{oc}}} = \frac{{mk_{\mathrm{B}}T}}{e}{\mathrm{ln}}\left( {\frac{{J_{{\mathrm{sc}}}}}{{J_0}} + 1} \right).$$

In principle, *m* = 1 represents direct bimolecular recombination. *m* = 2 indicates a Shockley–Read–Hall (SRH) recombination dominated, which means indirect recombination by trapping of minority carriers.

As shown in Fig. 5d, the obtained *J*_0_ of APSA-treated MAMP device was around one order of magnitude smaller than that of BA and pristine MAMP device. The lower *J*_0_ proved that lower shallower defect states existed in the system, which was in good agreement with previous measurement. Ideality factor was extracted by estimating the slope of the semilogarithmic current density–voltage (*J–V*) curve in the diffusion-dominated current region. As shown in Fig. 5d, the ideality factor was determined to be 1.48 for APSA-treated MAMP device, which was the lowest value among the devices. The same trend could also be demonstrated by *V*_oc_ evolution for different illumination intensities (Supplementary Fig. 26). A low ideality factor indicated that trap-assistant SRH recombination was suppressed in the homogeneous energy landscape film. Furthermore, electrochemical impedance spectroscopy demonstrated that APSA-treated MAMP device possessed the lowest charge transport resistance (*R*_ct_), and the highest charge recombination resistance (*R*_rec_) (Supplementary Fig. 27). The charge transport lifetime (*τ*_tr_) and the charge recombination lifetime (*τ*_rec_) were estimated from the transient photocurrent and transient photovoltage measurements, respectively (Supplementary Fig. 28). APSA-treated MAMP device showed faster photocurrent decay but slower photovoltage decay than the other two analogs, indicating that homogeneous energy landscape did improve the carrier transport and reduce the recombination.

In the next step, to quantitatively evaluate and correlate the recombination rate with the *V*_oc_ and device performance, we rewrite the equation based on a balance analysis of absorbed and emitted photons^36^. We then found

6 $$V_{\mathrm{oc}}=\frac{K_{\mathrm{B}}T}{e} {\mathrm{In}}\left({\mathrm{EQE}}_{\mathrm{EL}}\frac{J_{\mathrm{photo}}}{J_{\mathrm{dark}}}+1\right) ≈\, V_{\mathrm{oc,rad}}-\frac{K_{\mathrm{B}}T}{e}{\mathrm{In}}{\mathrm{{EQE}}_{\mathrm{EL}}^{-1}}.$$

The second term refers to the *V*_oc_ loss induced by the nonradiative recombination. The *V*_oc_ loss can be determined through the measurement of electroluminescence EQE (EQE_EL_) of quasi-2D perovskite devices (Fig. 5e). Compared with BA and pristine MAMP devices, APSA-treated MAMP device exhibited the highest EQE_EL_ of 0.15%. By consideration the Shockley-Queisser limit, 1.40 V is the theoretical radiative limit of *V*_oc_ for quasi-2D perovskites with a bandgap of 1.68 eV^37^. Attainable *V*_oc_ for APSA-treated MAMP perovskite should be around 1.23 V, which is close to our experimental data. Hence, various electrical and optical measurements confirmed that the homogeneous energy landscape did significantly reduce the energy disorder and decrease the defect states. The subsequently reduced band tail states and decreased SRH recombination lead to more ideal diode behaviors, which could be directly responsible for minimal *V*_oc_ loss.

To correlate the relationship between energy-level homogeneity and device performance, SCAPS simulation (Supplementary Table 4 and Supplementary Note 2) was carried to simulate device performance for MAMP devices with different energy disorder^38^, using the measured experimental optoelectronic parameters. As shown in Fig. 5f, SCAPS simulation revealed that the increased energy disorder significantly caused the *V*_oc_ deficit, accompanied by the FF shrinking and a decreased PCE, which reflected the same trend with our experimental data.

We then evaluated the stability of unencapsulated devices under conditions of 40–50% relative humidity. As shown in Fig. 5g, the control device, BA perovskite that was fabricated by the traditional hot-casting method (Supplementary Fig. 19b), underwent a significant degradation, which only retained 68% of its original PCE after 1000 h. This was not surprising since the high *<n*> value phases tended to stay at the top, according to previous discussion. The high *<n*> value species were not robust enough, which was responsible for the faster degradation. On the contrary, low *<n*> value species tended to stay on the top in APSA-treated MAMP film. The resulting device displayed negligible degradation under the same atmosphere, retaining almost 92% PCE after 1000 h. Even though the *<n*> value distribution was very narrow, the robust low *<n*> value species still showed advantages and provided better protection to humidity. We also tracked the light and thermal stability of the devices (Supplementary Fig. 29). APSA-treated MAMP devices still maintained more than 90% of their initial PCE, under 1-Sun illumination for 505 h, or under 80 °C heat treatment for 217 h. Moreover, X-ray diffraction patterns also confirmed that APSA-treated MAMP films exhibited better stability than BA ones under humidity, light, and thermal environments (Supplementary Fig. 30). As shown, lead iodide peaks were observed in the aged BA film. In contrast, no significant change was observed for APSA-treated MAMP film. It has been proven that DJ-phase quasi-2D perovskites typically exhibit stronger rigidity and interlayer connection than Ruddlesden-Popper-phase ones^39^.

## Discussion

In summary, we have demonstrated that the homogeneous energy landscape is essential to achieve high-performance quasi-2D perovskite photovoltaics, especially high *V*_oc_. Toward this goal, we conceive synergistic strategy to accomplish homogeneous energy landscape, by taking advantage of material structure and crystallization kinetics engineering. With the help of the DFT-guided material design, minimization of the band offsets has been achieved in MAMP-based quasi-2D perovskite, which results in the lowest energy distribution in the SC state to date. On top of this, we further regulate the crystallization kinetics to realize a narrow <*n*> value distribution for MAMP film. As a result, a homogeneous energy landscape is formed in APSA-treated MAMP film. We correlate the relationship between the energy landscape homogeneity and device performance, and further investigate the physical origin of low *V*_loss_. Owing to the reduced nonradiative recombination induced by the homogeneous energy landscape, the device achieves an excellent PCE of 16.53%, accompanied by a high *V*_oc_ of 1.21 V. In addition, the device exhibits significantly improved stability compared with traditional hot-casting films, due to the more ideal vertical phase alignment and absent high <*n*> value phases. As a result, APSA-treated MAMP retains almost 92% of the initial PCE after 1000 h of storage.

## Methods

### Materials

All chemicals were obtained from Sigma-Aldrich and Lumtec Corp without further purification. TiO_2_ nanocrystals were synthesized according to the previous procedure^40^.

### Perovskite SC synthesis

BA_2_MA_*n*−1_Pb_*n*_I_3*n*+1_ SCs were grown by using an improved cooling method. Briefly, mixed PbO, MAI, and BAI at different ratios were dissolved in hydroiodic acid (HI) solution at  about 90 °C. The resulting solution was filtered and gradually cooled down to 20 °C at a rate of 1 °C h^−1^. For MAMPMA_*n*−1_Pb_*n*_I_3*n*+1_ SCs, different ratios of PbO, MAI, and MAMPI mixture were dissolved in 30 mL of HI solution at about 110 °C. The resulting solution cooled down to 20 °C at a rate of 0.5 °C h^−1^. As-grown perovskite crystals were wiped and rinsed with diethyl ether to remove the surface residues.

### Device fabrication

The cleaned ITO glass substrates were treated using an ultraviolet zone for 10 min prior to remove the organic residues. TiO_2_ nanocrystals were spin-coated on ITO substrates at 3000 r.p.m. for 30 s, and annealed for 30 min at 150 °C. The substrates were then transferred to nitrogen-filled glovebox to deposit perovskite absorbers. The corresponding precursors for (BA)_2_MA_3_Pb_4_I_13_ and pristine (MAMP)MA_3_Pb_4_I_13_ were prepared and weighted at the stoichiometric ratio. The mixtures were dissolved in the mixed solvent, DMF:DMSO (dimethylformamide:dimethyl sulfoxide), which equals 7:3, to set the final concentration at 1.4 M. For APSA-treated (MAMP)MA_3_Pb_4_I_13_ film, APSA was directly introduced into the pristine (MAMP)MA_3_Pb_4_I_13_ perovskite solution with different concentrations. Accordingly, the precursor solutions were spin-coated on top of the TiO_2_ film at 4000 r.p.m. for 50 s, and 200 μl of ethyl acetate was dropped onto the film during the process, followed by annealing process at 130 °C for 15 min. The prepared Spiro-OMeTAD solution was then deposited at 4000 r.p.m. for 30 s. Finally, about 100 nm of gold electrode was thermally evaporated on the top of films at a pressure of 5 × 10^−6^ mbar.

### Photovoltaic device characterization

The steady-state *V*_oc_, was first measured using a Keithley 2400 instrument by fixing the current to 0, and sampling the voltage at multiple time points. The steady-state *J*_sc_, was measured via setting the bias voltage to 0, and sampling the current at multiple time points. Then the instantaneous *J–V* curves were measured at a scanning rate of 50 mV s^−1^ with delay time of 200 ms and voltage step of 10 mV. The active area of 0.049 cm^2^ was determined by an aperture, and the output of the light source was adjusted via a calibrated silicon photodiode (Newport) at 1 Sun (100 mW cm^−2^). The AM 1.5 solar power was supplied by a class A solar simulator (Enli Tech) with the mismatch factor less than 25%. The spectral mismatch factor (less than 5%) of the system was characterized using a calibrated reference solar cell (Newport). The EQE spectra of devices were obtained following a previously reported process.

### DLS characterization

DLS measurements were performed by a Zetasizer Nano ZS instrument (Malvern Instruments, UK) with a 633-nm He–Ne laser. The perovskite precursor solutions are transferred into the quartz cuvettes and sealed in a N_2_ glovebox. The measurement was done at room temperature.

### GIWAXS characterization

GIWAXS patterns of quasi-2D perovskite films were measured on the beamline 1W2A at Beijing Synchrotron Radiation Facility (BSRF), China. A monochromatic beam (1.54 Å) was used at 0.1° and 0.3° of grazing-incidence angles. All films were measured via the same exposure time of 240 s. GIWAXS patterns were collected at a beam energy of 10 keV by an area detector with 3450 × 3450-pixel resolution. GIWAXS raw patterns were corrected by GIXSGUI MATLAB toolbox.

### The ultrafast TA spectroscopy characterization

TA measurements of quasi-2D films were carried out by a Helios pump–probe system (Ultrafast Systems LLC) with an amplified femtosecond laser system (Coherent, 35 fs, 7 mJ per pulse, 1 kHz, 800 nm). Thepump pulses (365 nm, 8 nJ per pulse) were generated by an optical parametric amplifier (TOPAS-800-fs). Focusing the 800-nm beams (split from the regenerative amplifier with a tiny portion, 400 nJ per pulse) onto a sapphire plate produced the white-light continuum (WLC) probe pulses. The pulse-to-pulse fluctuation of the WLC was corrected by a reference beam split from WLC. A motorized optical delay line was served as a tool to change the time delays between pump and probe pulses. The instrument response function (100 fs) was determined via a routine cross-correlation procedure. A mechanical chopper (500 Hz) modulated the pump pulses. Temporal and spectral profiles (chirp-corrected) of the WLC probe light were visualized through the optical fiber-coupled multichannel spectrometer [with a CMOS (complementary metal oxide semiconductor) sensor] and the Surface Xplorer software.

### Reporting summary

Further information on research design is available in the Nature Research Reporting Summary linked to this article.

## Supplementary information

Supplementary Information

Reporting Summary

## Data Availability

All data generated or analyzed during this study are included in this published article and its supplementary information files.
