# Supplementary material for: Reduced-dimensional perovskite photovoltaics with homogeneous energy landscape
Source: Nat Commun. 2020 Apr 3;11:1672. doi: 10.1038/s41467-020-15451-1 (PMC7125147; doi:10.1038/s41467-020-15451-1)
Supplement: Supplementary file 1 — Supplementary Information [file 41467_2020_15451_MOESM1_ESM.pdf]

Supplementary Information

Reduced-Dimensional Perovskite Photovoltaics with Homogeneous Energy Landscape

He et al.

Supplementary Figures

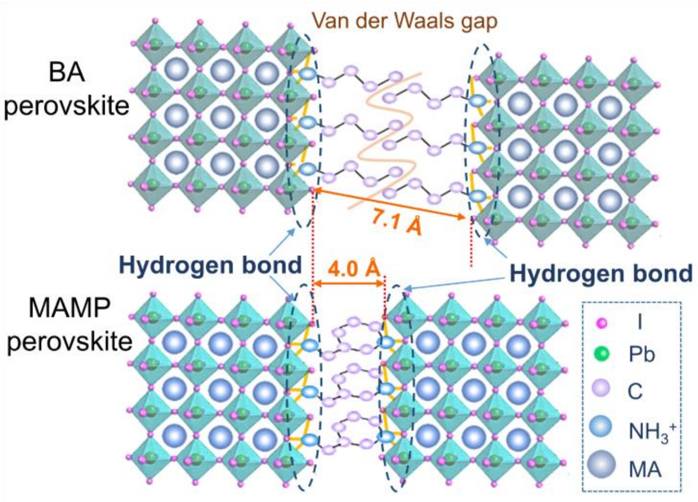

Supplementary Figure 1 | Schematic diagram for crystal structure of $(BA)_2MA_3Pb_4I_{13}$ and $(MAMP)MA_3Pb_4I_{13}$ perovskite. The crystal structure was derived from the crystallographic data reported by Kanatzidis *et al*.[1,2] BA: *n*-butylammonium, $CH_3(CH_2)_3NH_2$; MAMP: *meta*-(aminomethyl) piperidinium, $NH_2CH_3C_5NH_{12}$. The measured interlayer I-I distance for $(MAMP)MA_3Pb_4I_{13}$ and $(BA)_2MA_3Pb_4I_{13}$ determined to be about 4.0 Å and 7.1 Å, respectively.

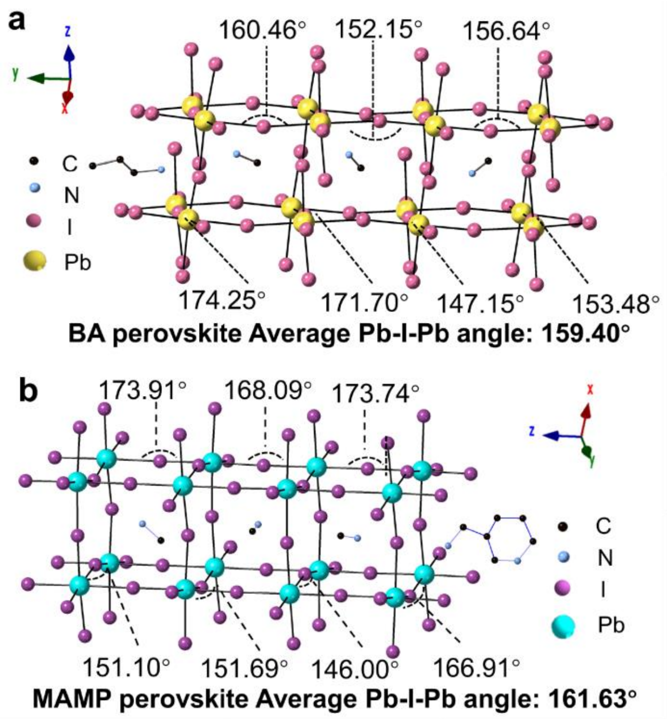

Supplementary Figure 2 | The calculated average Pb-I-Pb angles for **a** $(BA)_2(MA)_3Pb_4I_{13}$ and **b** $(MAMP)(MA)_3Pb_4I_{13}$ perovskites.

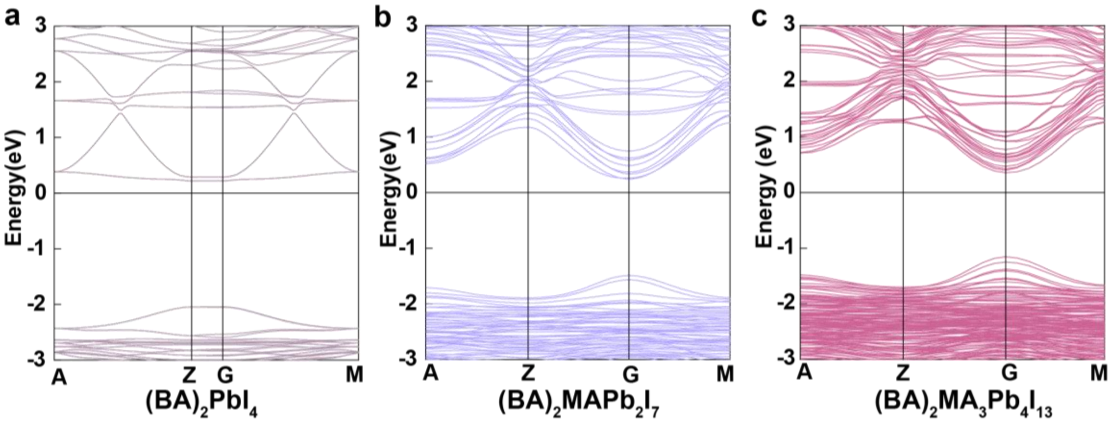

Supplementary Figure 3 | Band structure for **a** $(BA)_2PbI_4$, **b** $(BA)_2MAPb_2I_7$ and **c** $(MAMP)MA_3Pb_4I_{13}$ perovskites derived from DFT simulation. The simulated bandgap is 2.23 eV and 1.72 eV and 1.47 eV, respectively.

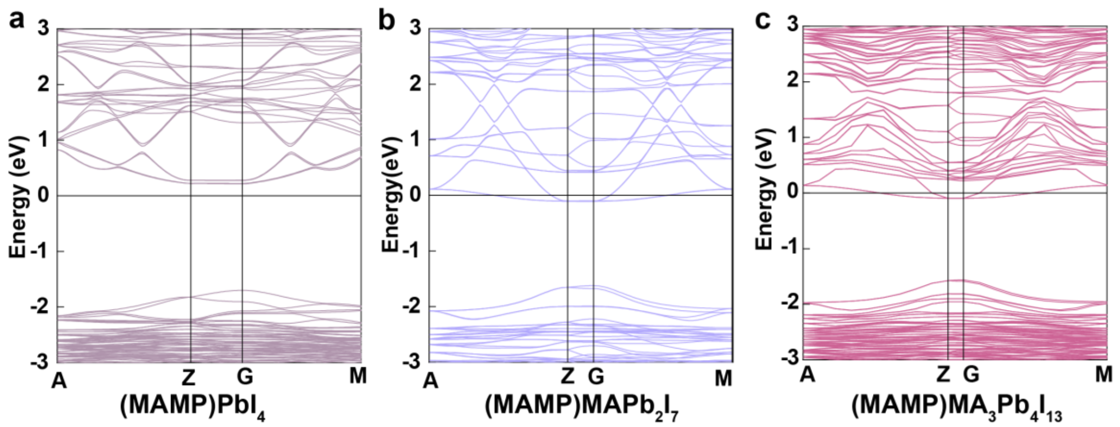

Supplementary Figure 4 | Band structure for **a** (MAMP)PbI_4, **b** (MAMP)$MAPb_2I_7$ and **c** (MAMP)$MA_3Pb_4I_{13}$ perovskites derived from DFT simulation**.** The determined bandgap is 1.91 eV, 1.51 eV and 1.42 eV, respectively.

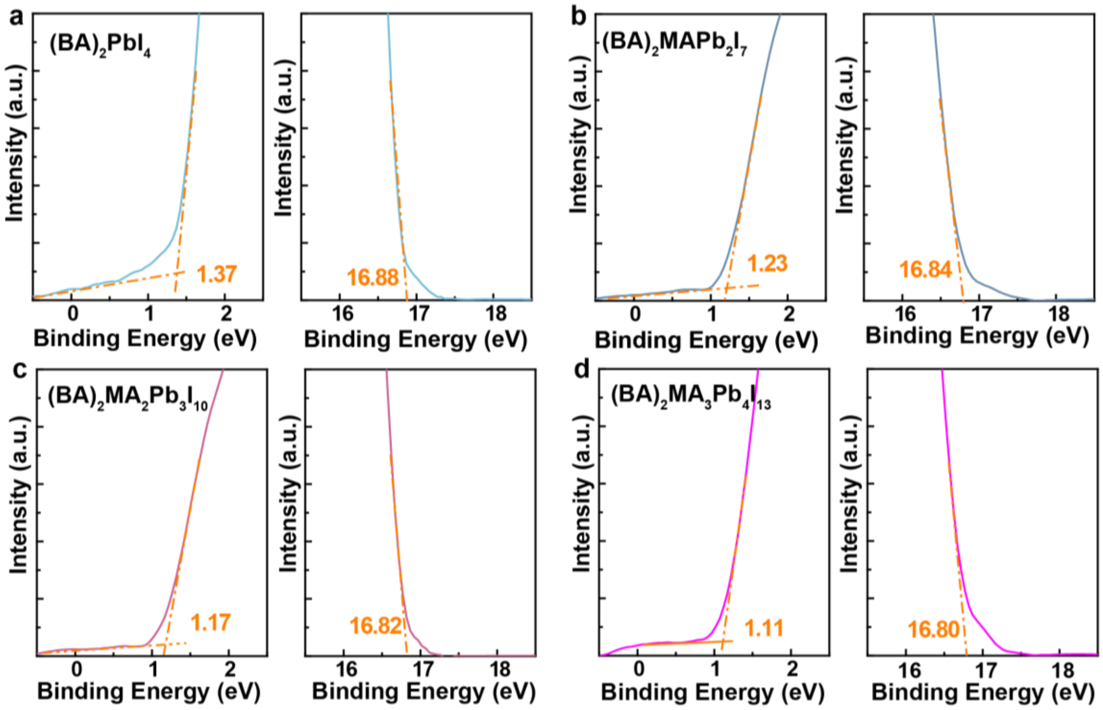

Supplementary Figure 5 | UPS characterization of the energy level for $(BA)_2MA_{n-1}Pb_nI_{3n+1}$ (*n* is 1, 2, 3, 4) perovskite single crystals.

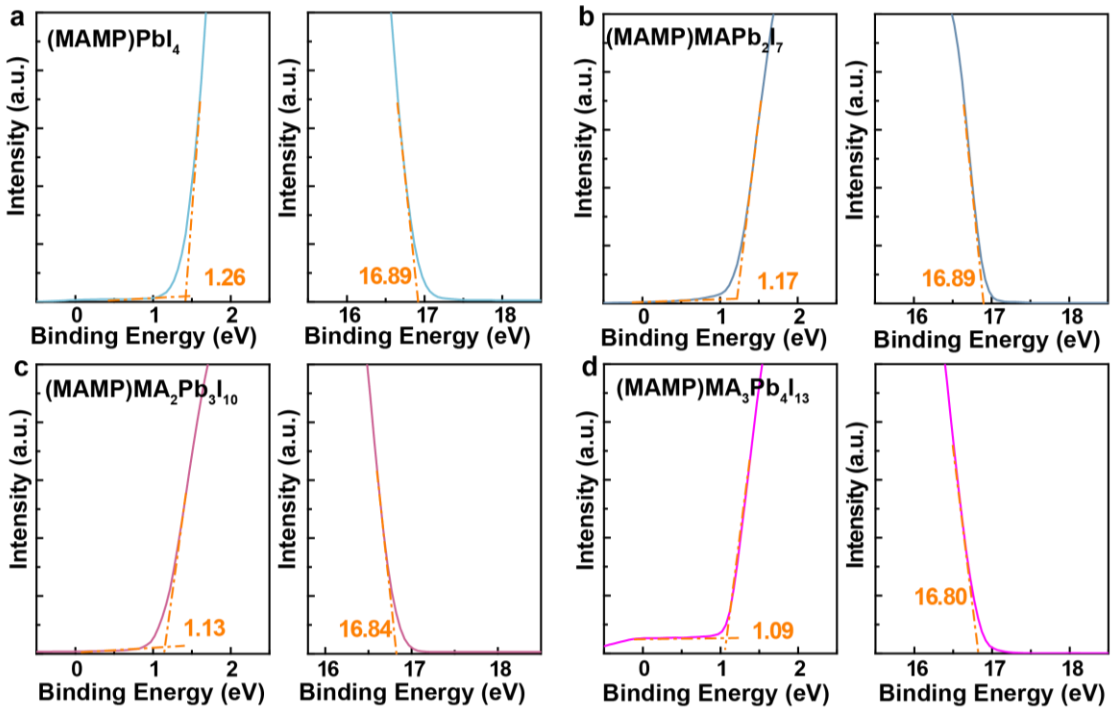

Supplementary Figure 6 | UPS characterization of the energy level for (MAMP)$MA_{n-1}Pb_nI_{3n+1}$ (*n* is 1, 2, 3, 4) perovskite single crystals.

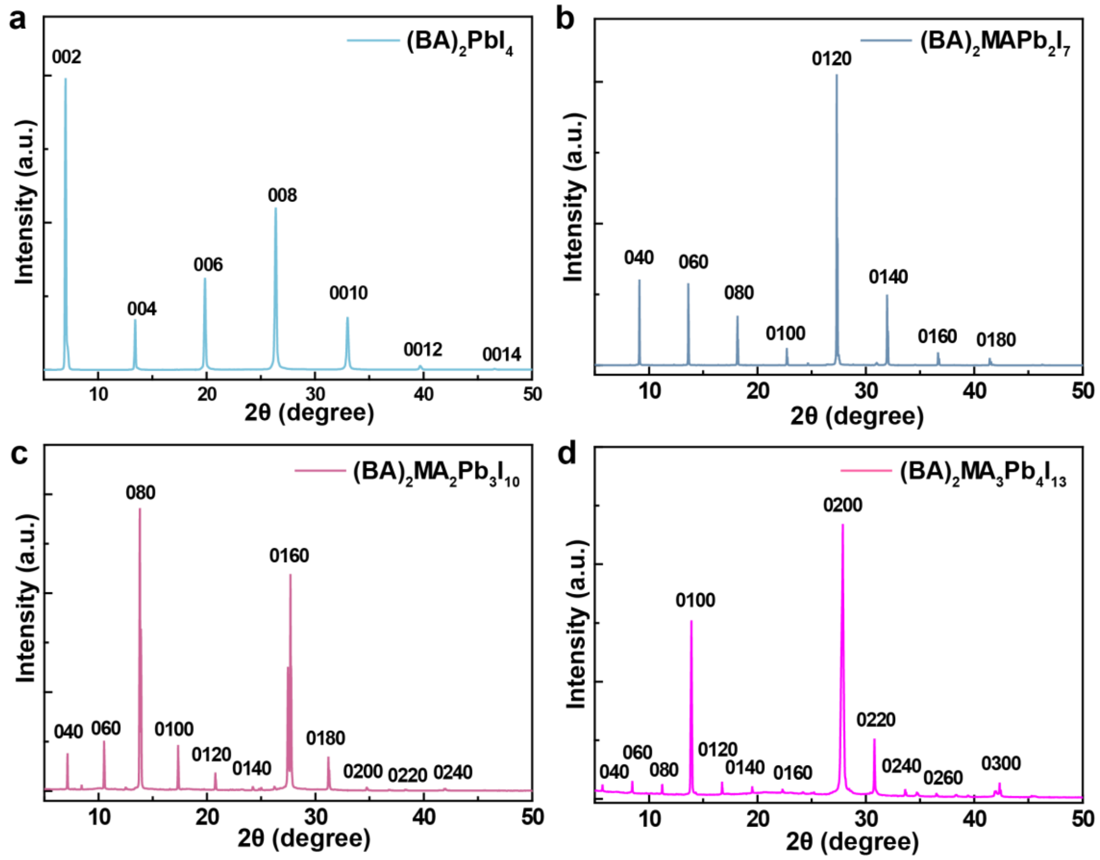

Supplementary Figure 7 | XRD patterns of the energy level for $(BA)_2MA_{n-1}Pb_nI_{3n+1}$ (n is 1, 2, 3, 4) perovskite single crystals.

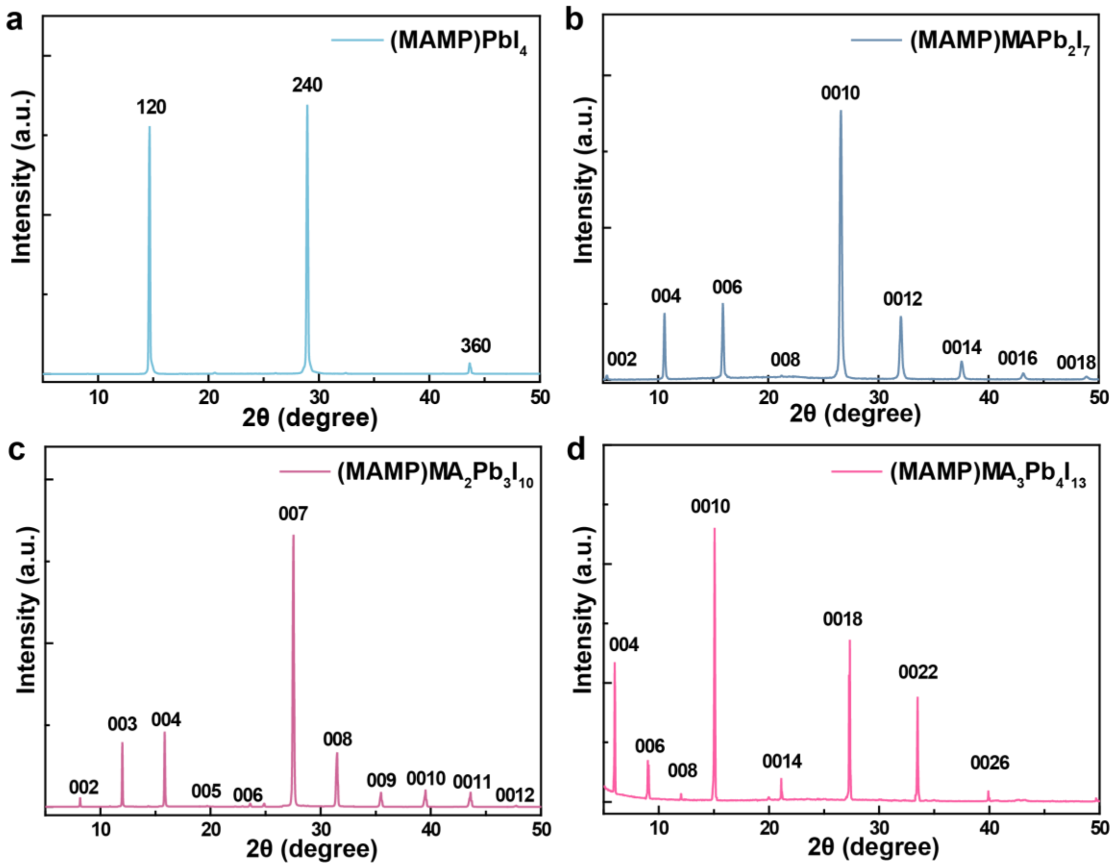

Supplementary Figure 8 | XRD patterns of the energy level for $(MAMP)_2MA_{n-1}Pb_nI_{3n+1}$ (n is 1, 2, 3, 4) perovskite single crystals.

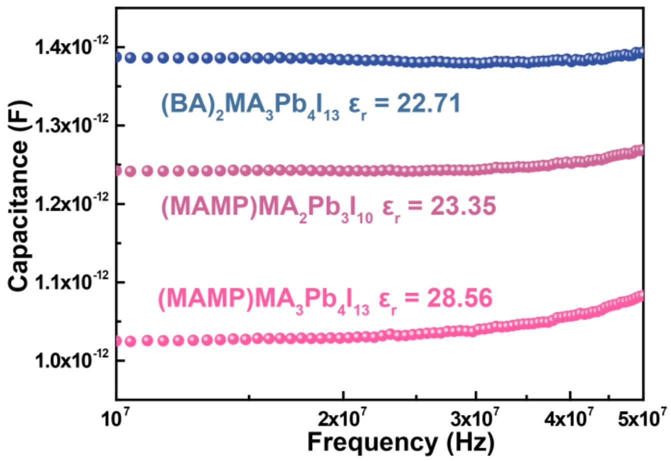

Supplementary Figure 9 | The frequency dependent capacitance curve of $(BA)_2MA_3Pb_4I_{13}$, $(MAMP)MA_2Pb_3I_{10}$ and $(MAMP)MA_3Pb_4I_{13}$ single crystals.

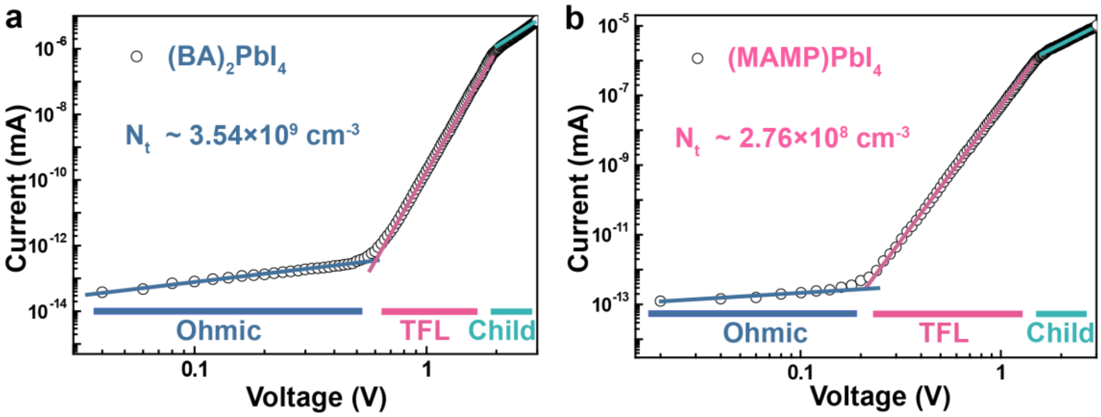

Supplementary Figure 10 | SCLC curves of **a** $(BA)_2PbI_4$ and **b** $(MAMP)PbI_4$ single crystals.

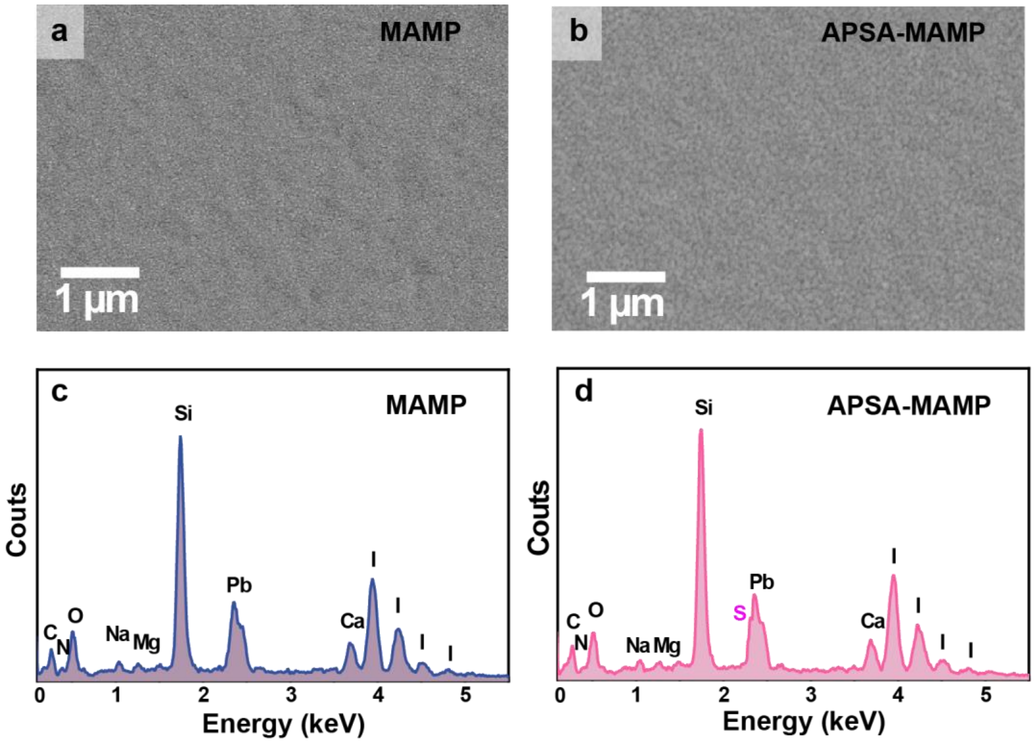

Supplementary Figure 11 | SEM images and corresponding EDS analysis for pristine and APSA treated (MAMP)$MA_3Pb_4I_{13}$ perovskite films.

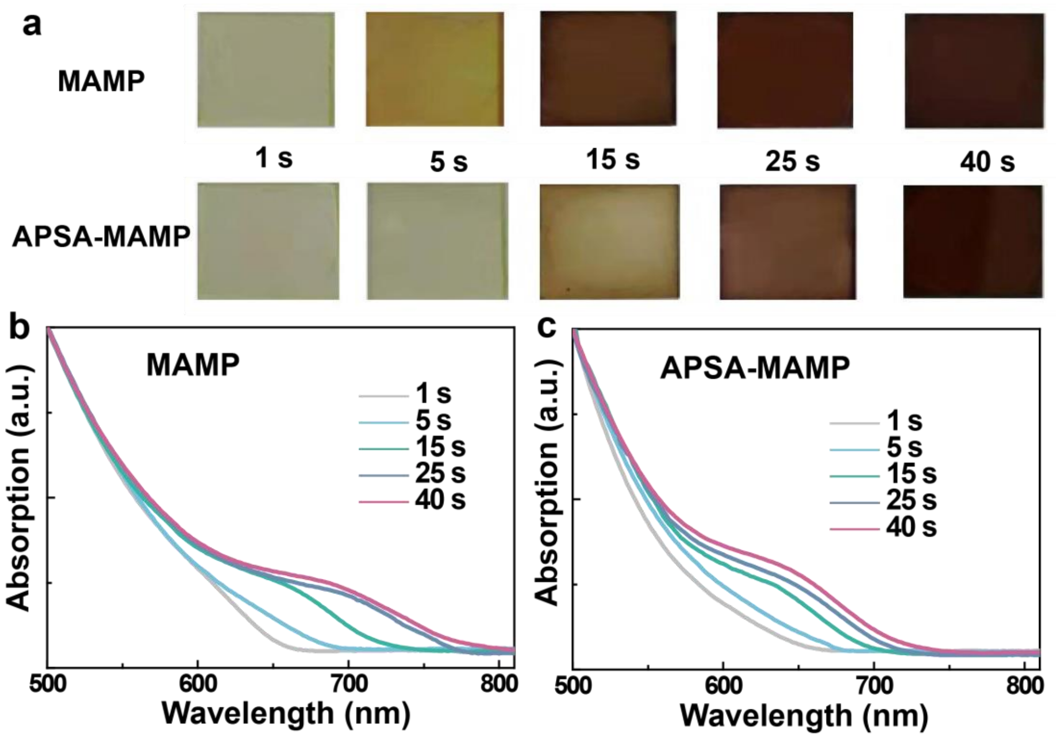

Supplementary Figure 12 | Optical images and resulting absorption spectra of pristine and APSA treated $(MAMP)MA_3Pb_4I_{13}$ perovskite films, at different timescale.

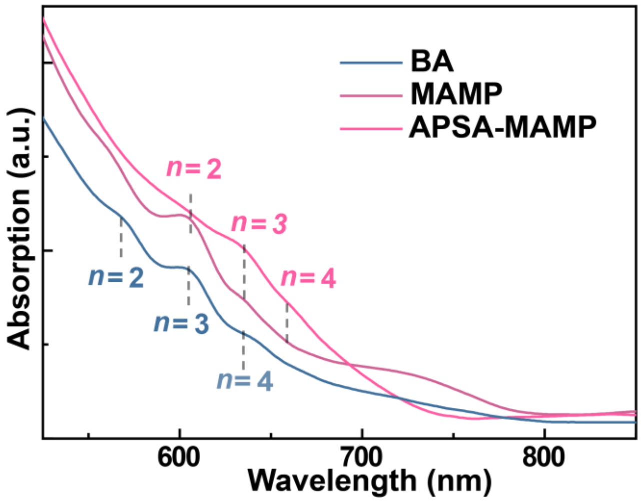

Supplementary Figure 13 | Absorption spectra of $(BA)_2MA_3Pb_4I_{13}$, pristine and APSA treated $(MAMP)MA_3Pb_4I_{13}$ perovskite films. A broad new peak arising in the long-wavelength region has been observed before ASPA treatment, which ascribes to the high <*n*>-values species.

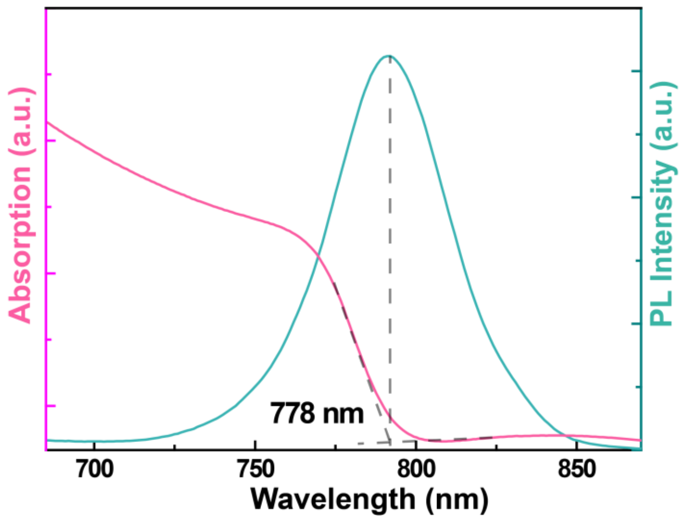

Supplementary Figure 14 | Absorption and steady-state PL spectra of $MAPbI_3$ perovskite films.

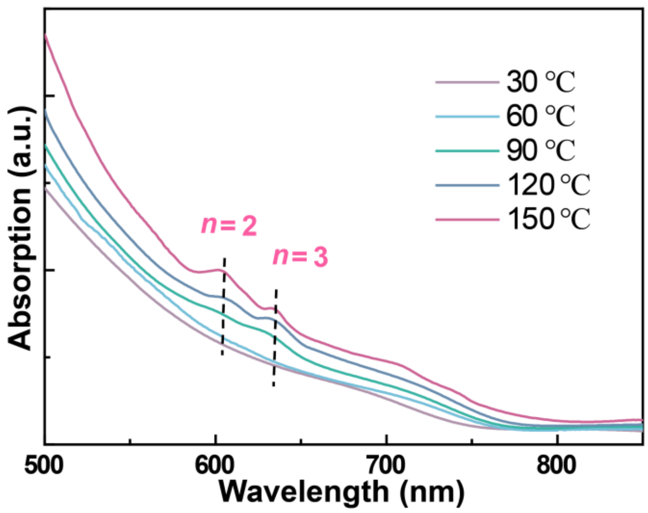

Supplementary Figure 15 | Absorption spectra of pristine (MAMP)$MA_3Pb_4I_{13}$ perovskite films at different annealing temperature.

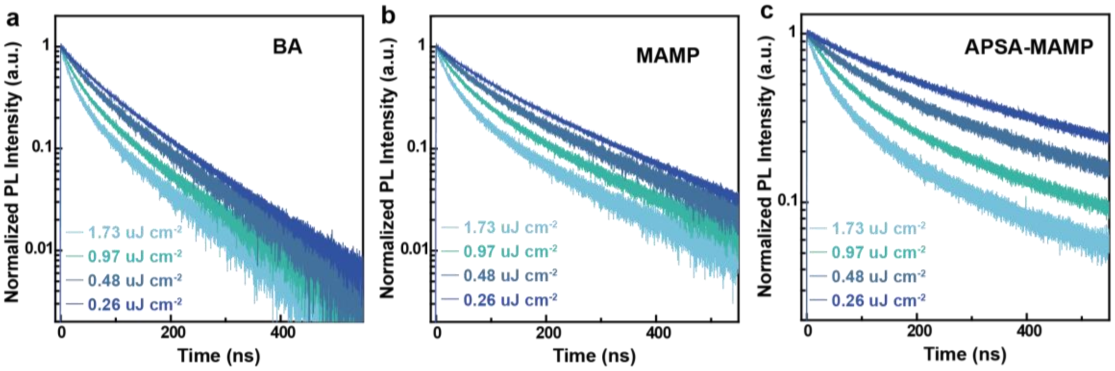

Supplementary Figure 16. Density dependent TRPL of **a** $(BA)_2MA_3Pb_4I_{13}$, **b** pristine and **c** APSA treated $(MAMP)MA_3Pb_4I_{13}$ perovskite films.

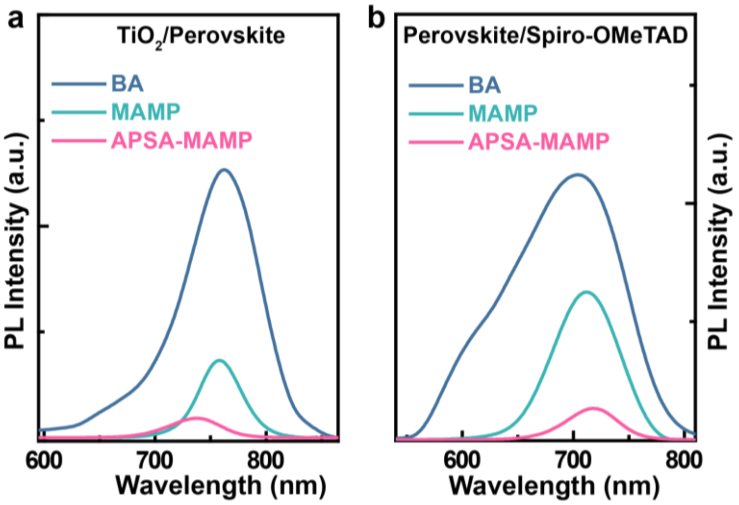

Supplementary Figure 17 | The steady-state PL spectra of BA film, pristine MAMP film and APAS treated MAMP film based on the **a** electron transport layer, and **b** hole transfer layer.

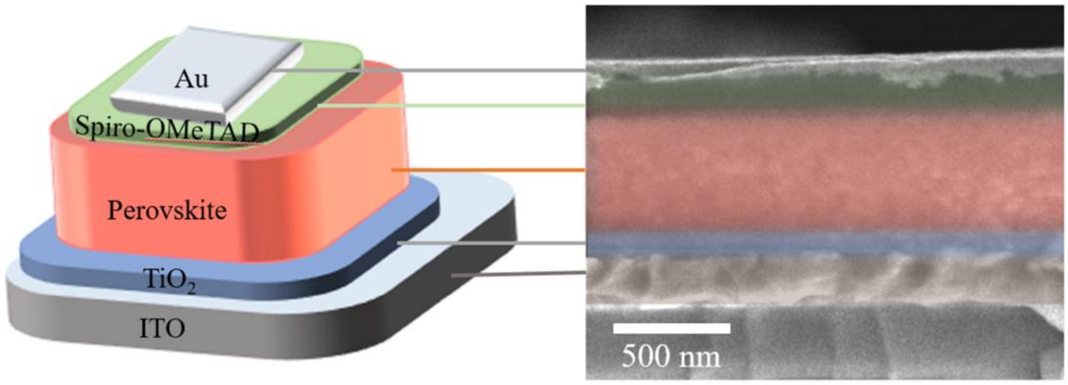

Supplementary Figure 18 | Schematic diagram of conventional device architecture applied, and the cross-section SEM image for the ASPA treated (MAMP)$MA_3Pb_4I_{13}$ quasi-2D perovskite photovoltaics.

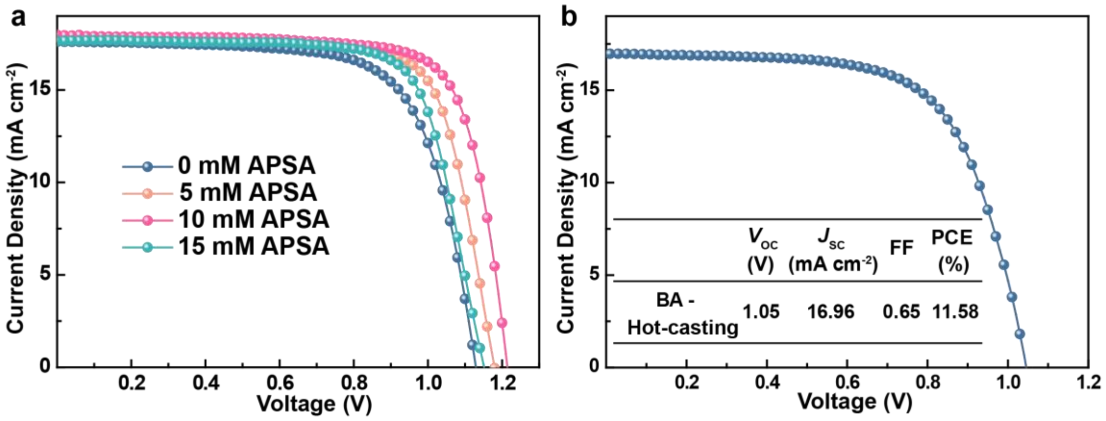

Supplementary Figure 19 | a *J-V* curves of (MAMP)$MA_3Pb_4I_{13}$ quasi-2D perovskite photovoltaics with different amount of APSA treatment; **b** Device performance for the control $(BA)_2MA_3Pb_4I_{13}$ quasi-2D perovskite photovoltaics with an inverted device architecture.

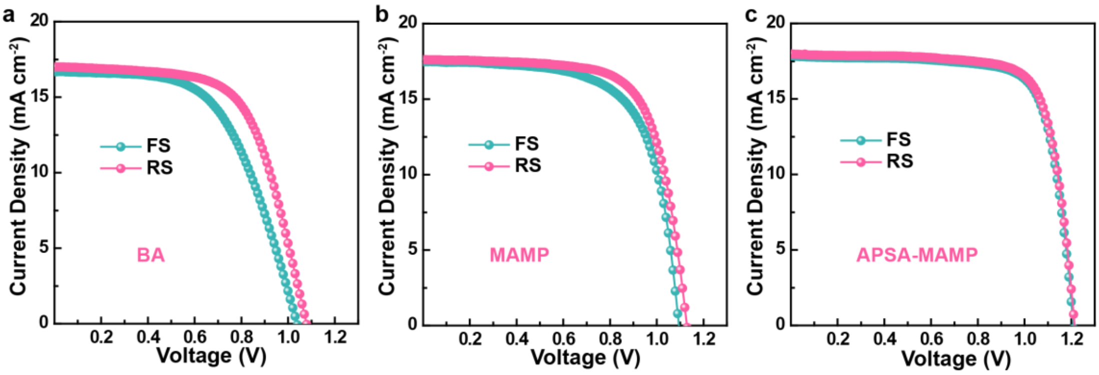

Supplementary Figure 20 | The *J*-*V* curves of $(BA)_2MA_3Pb_4I_{13}$, pristine $(MAMP)MA_3Pb_4I_{13}$ and APSA treated $(MAMP)MA_3Pb_4I_{13}$ perovskite photovoltaics for different scanning directions.

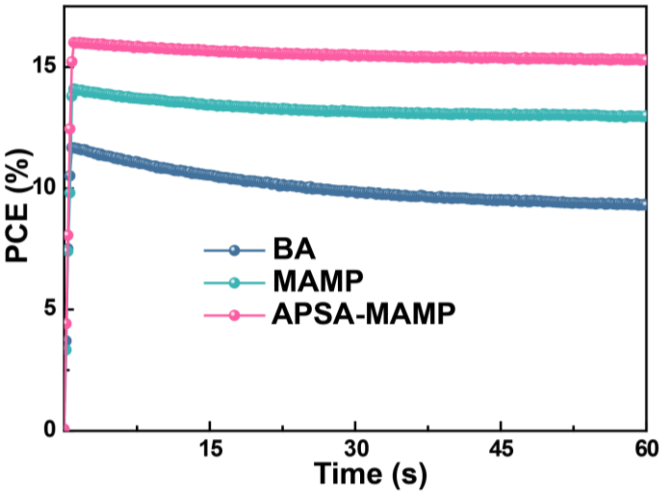

Supplementary Figure 21 | Stabilized power output of three champion cells a fixed voltage near the maximum power point on the *J-V* curve for 60 s.

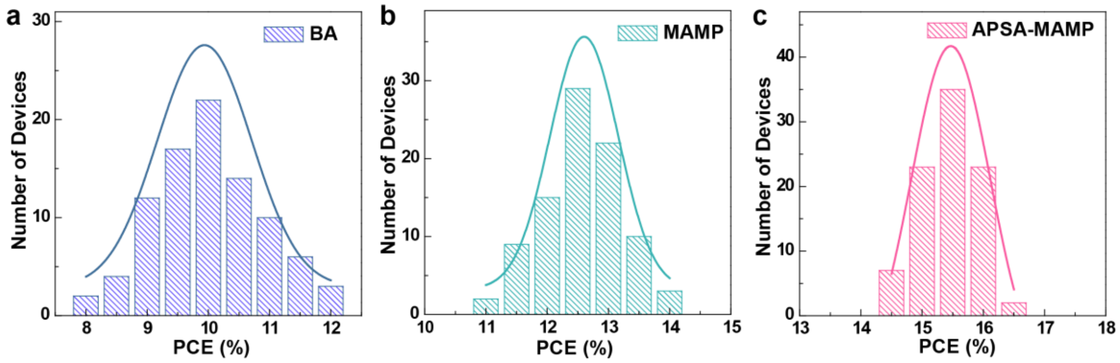

Supplementary Figure 22 | The histograms of device performance for $(BA)_2MA_3Pb_4I_{13}$, pristine $(MAMP)MA_3Pb_4I_{13}$ and APSA treated $(MAMP)MA_3Pb_4I_{13}$ perovskite photovoltaics.

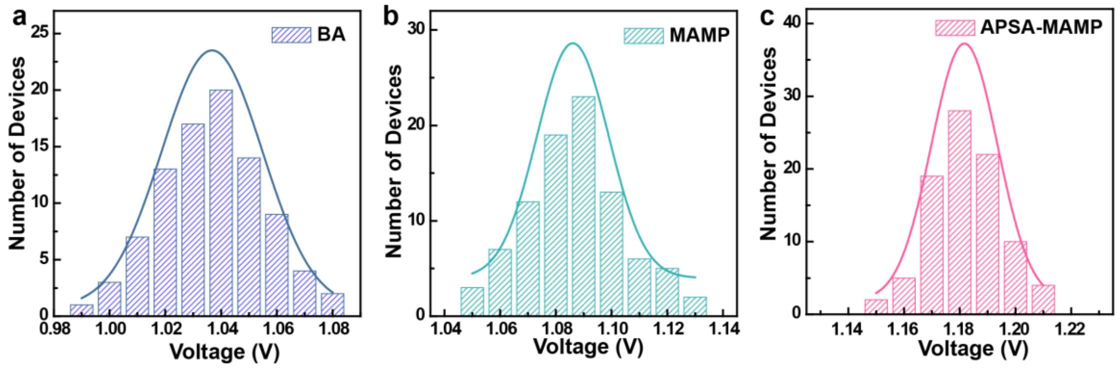

Supplementary Figure 23 | The histograms of V_{oc} for $(BA)_2MA_3Pb_4I_{13}$, pristine $(MAMP)MA_3Pb_4I_{13}$ and APSA treated $(MAMP)MA_3Pb_4I_{13}$ perovskite photovoltaics.

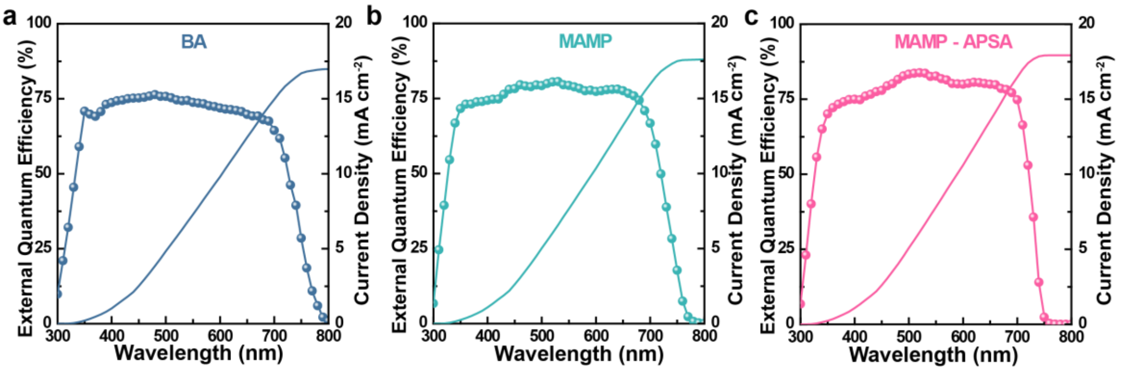

Supplementary Figure 24. EQE spectra of **a** $(BA)_2(MA)_3Pb_4I_{13}$, **b** pristine and **c** APSA treated $(MAMP)MA_3Pb_4I_{13}$ devices.

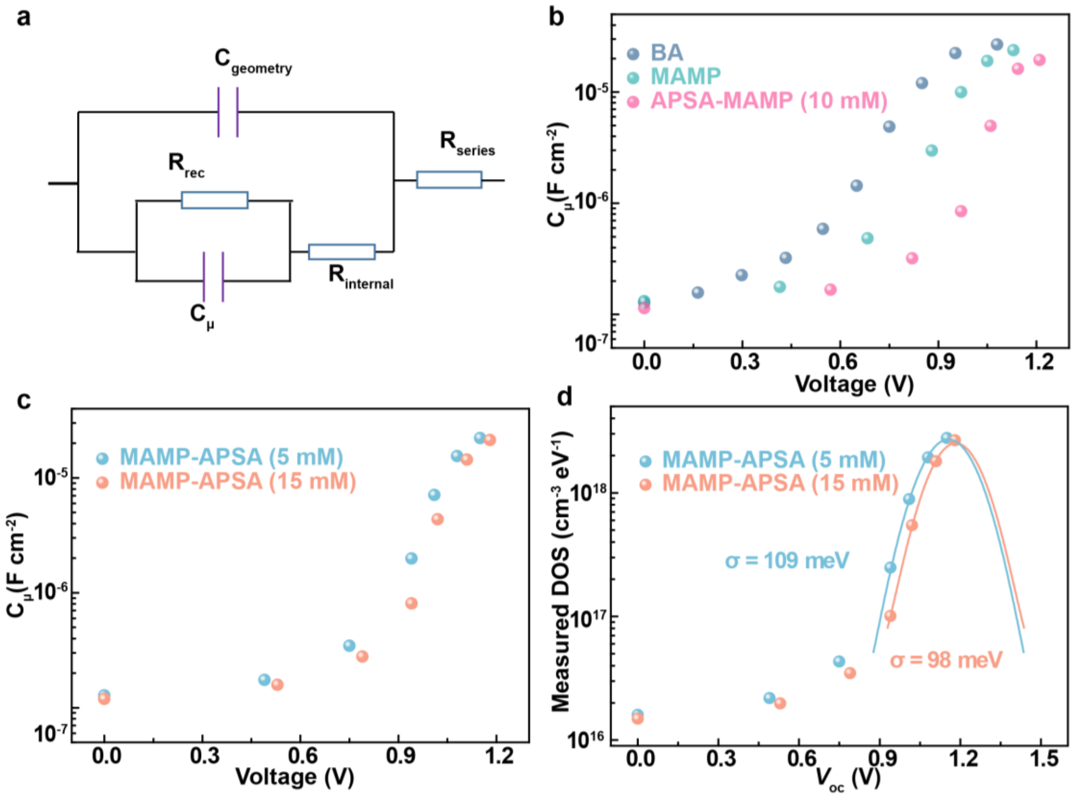

Supplementary Figure 25 | Energetic disorder parameter extracted from impedance spectroscopy. **a** equivalent circuit illustrate the impedance spectroscopy measurement. **b** Chemical capacitance that extracting from impedance spectra for $(BA)_2(MA)_3Pb_4I_{13}$, pristine $(MAMP)MA_3Pb_4I_{13}$ and 10 mM APSA treated $(MAMP)MA_3Pb_4I_{13}$ perovskite, respectively. **c** Chemical capacitance and **d** DOS for $(MAMP)MA_3Pb_4I_{13}$ perovskite treated with different amount of ASPA. The applied bias voltage equaled the V_{oc} of device under each light intensity. $R_{internal}$, R_{rec}, $C_{geometry}$ and C_{μ} are the internal resistance, the recombination resistance, the geometry capacitance, and the chemical capacitance, respectively. C_{μ} follows the shape of the electron DOS (g_n)[3] as: $C_{\mu}{}^{(n)} = Lq^2 g_n(E_{Fn})$, DOS results are then fitted by Gauss function.

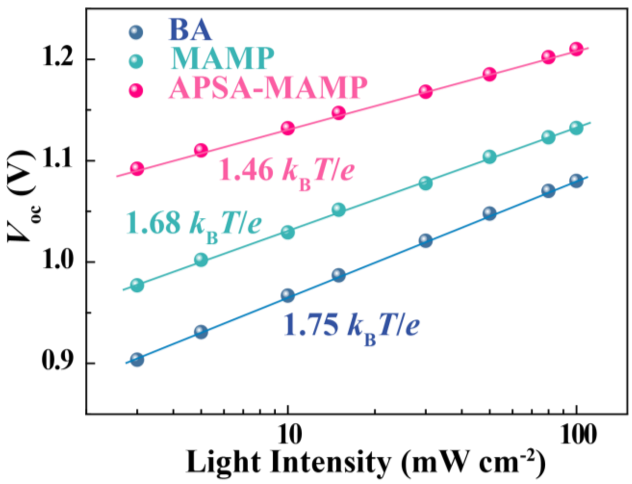

Supplementary Figure 26 | V_{oc} evolution of the devices under different illumination intensities.

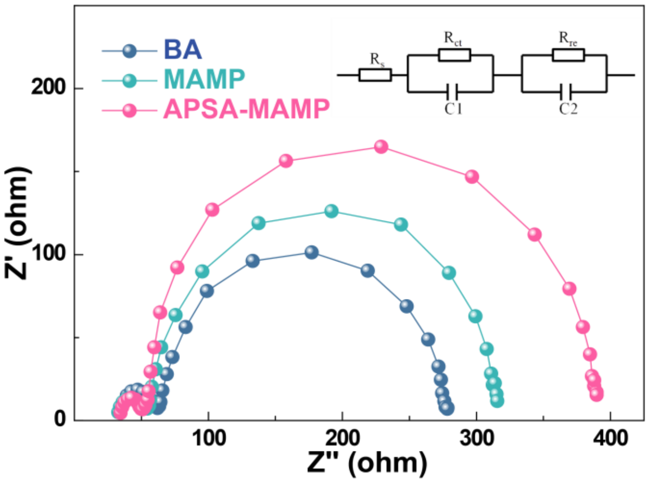

Supplementary Figure 27 | EIS spectra for three different perovskite devices.

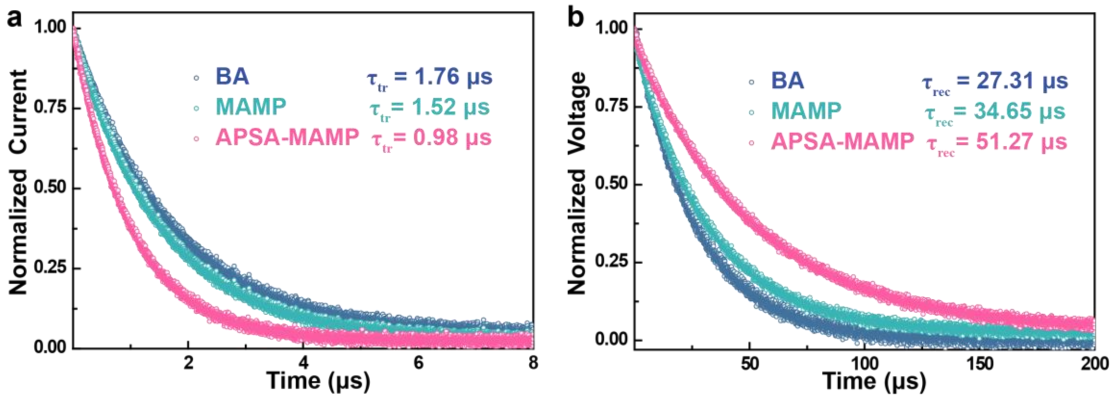

Supplementary Figure 28 | TPC and TPV measurements for $(BA)_2(MA)_3Pb_4I_{13}$, pristine $(MAMP)MA_3Pb_4I_{13}$ and APSA treated $(MAMP)MA_3Pb_4I_{13}$ perovskite photovoltaics.

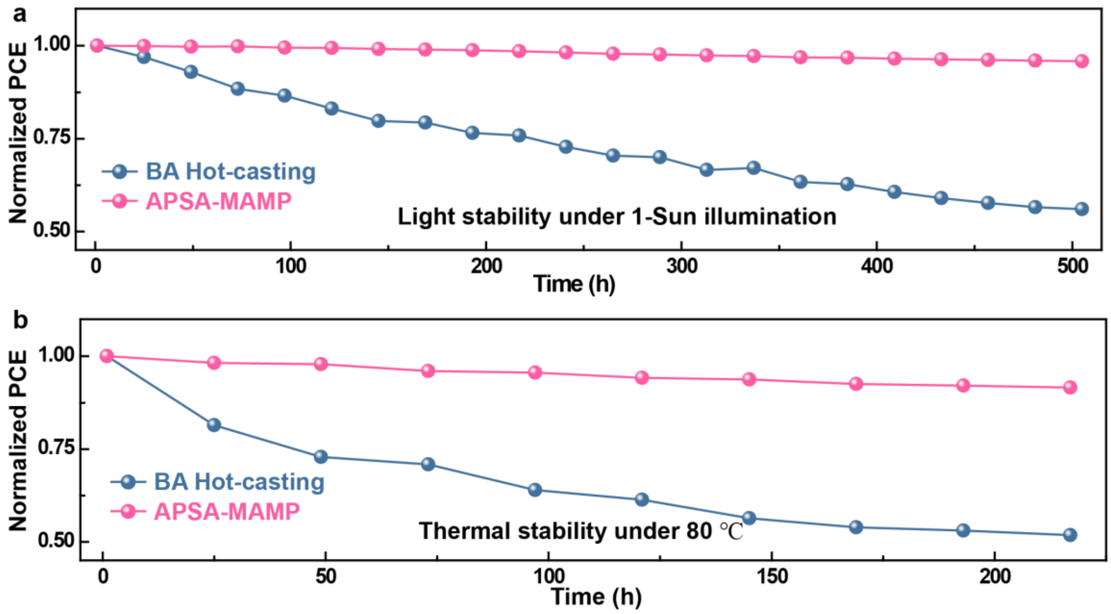

Supplementary Figure 29 | PCE evolution curves for unencapsulated ASPA treated $(MAMP)MA_3Pb_4I_{13}$ devices, and the $(BA)_2MA_3Pb_4I_{13}$ devices fabricated by traditional hot-casting method, under **a** 1-Sun illumination and **b** 80 ℃ in air.

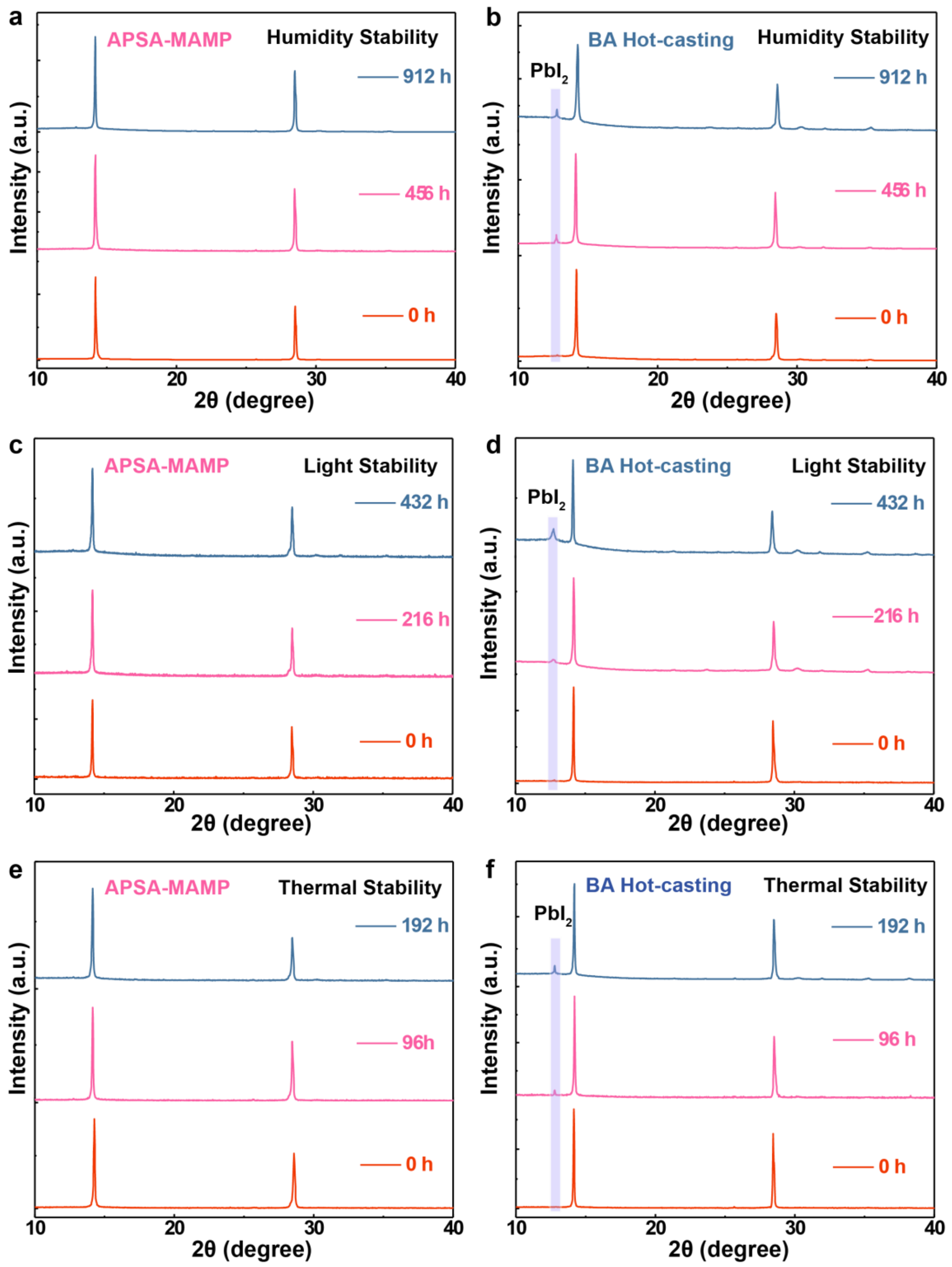

Supplementary Figure 30 | XRD Patterns of ASPA treated $(MAMP)MA_3Pb_4I_{13}$ film and the $(BA)_2MA_3Pb_4I_{13}$ film fabricated by traditional hot-casting method, under **a, b** humidity of 40% to 50% RH, **c, d** 1-Sun illumination and **e, f** 80 ℃ in air.

Supplementary Tables

Supplementary Table 1 | VBM and CBM energy levels of $(BA)_2MA_{n-1}Pb_nI_{3n+1}$ perovskite single crystals through UPS measurement.

***n* value**	**1**	**2**	**3**	**4**
E_g (eV)	2.44	2.15	2.03	1.92
VBM (eV)	5.71	5.61	5.57	5.53
CBM (eV)	3.27	3.46	3.54	3.61

Supplementary Table 2 | VBM and CBM energy levels of $MAMPMA_{n-1}Pb_nI_{3n+1}$ perovskite single crystals through UPS measurement.

***n* value**	**1**	**2**	**3**	**4**
E_g (eV)	2.22	2.05	1.96	1.89
VBM (eV)	5.59	5.53	5.51	5.49
CBM (eV)	3.37	3.48	3.55	3.60

Supplementary Table 3 | Photovoltaic parameters and hysteresis index of $(BA)_2MA_3Pb_4I_{13}$, pristine $(MAMP)MA_3Pb_4I_{13}$ and APSA treated $(MAMP)MA_3Pb_4I_{13}$ perovskite photovoltaics. Photovoltaic parameters and hysteresis index (*HI*) was calculated by $(\mathrm{PCE_{RS}} - \mathrm{PCE_{FS}})/\mathrm{PCE_{RS}}$.

Devices	Direction	V_{oc} (V)	J_{SC} (mA cm^{-2})	FF	PCE (%)	*HI*
BA	FS	1.03	16.69	0.57	9.88	0.16
	RS	1.08	17.01	0.64	11.76	
MAMP	FS	1.09	17.48	0.67	12.81	0.09
	RS	1.13	17.62	0.71	14.14	
APSA-MAMP	FS	1.20	17.84	0.76	16.34	0.01
	RS	1.21	17.97	0.77	16.53	

Supplementary Table 4 | Parameters of perovskite absorber layer for SCAPS simulation.

Symbol	Description	Parameter value
$L_{thickness}$	Thickness of absorber layer	500 nm
μ_e	Electron mobility	2.0 $cm^2 V^{-1} s^{-1}$ [4]
μ_h	Hole mobility	2.0 $cm^2 V^{-1} s^{-1}$ [4]
E_c	CB effective density of states	2.2×10^{18} cm^{-3} [5]
E_v	VB effective density of states	1.8×10^{19} cm^{-3} [5]
X_e	Electron affinity	3.9 eV [6]
E_g	Band gap of absorber	1.68 eV
ε_r	Relative dielectric constant	25 [7]
N_A	Self-Doping concentration (p-type)	5.0×10^{16} cm^{-3} [8]

Supplementary Notes

Supplementary Note 1 | Details for DFT Simulation.

The geometry optimization and electronic structure calculation were carried out using the Vienna Ab initio Simulation Package (VASP).[9] A uniform 4 × 4 × 2 Monkhorst−Pack k-point mesh and a plane-wave energy cutoff of 400 eV were adopted to the structural optimization. We used the Perdew−Burke−Ernzerhof (PBE) functional[10] to express the exchange-correlation interactions and projector-augmented wave (PAW) method[11] to account for the electron-ion interaction. The van der Waals interactions was described by the Grimme DFT-D3 correction.[12] Crystal models of $(BA)_2MA_{n-1}Pb_nI_{3n+1}$ and $(MAMP)MA_{n-1}Pb_nI_{3n+1}$ perovskites were derived from the literatures reported by Kanatzidis *et al.*[1,2]

To evaluate the distortion level at the individual $[PbI_6]^{4-}$ octahedron of MAMP and BA perovskite, we calculated the bond angle variance (BAV) in equation 1 and distortion index (DI) in equation 2 to quantize distortion from I-Pb-I angle and Pb-I bond length.

$$\delta^2 = \frac{\sum_{i=1}^{m}(\theta_i-\theta_0)^2}{m-1} \tag{1}$$

Here, m is the number of faces in the polyhedron × 1.5, θ_i is the ith bond angle, and θ_0 is the normal bond angle (90°) for a polyhedron.[13]

$$D = \frac{1}{n}\sum_{i=1}^{n}\frac{|I_i-I|}{I} \tag{2}$$

Here, Ii is the distance from the central atom to the ith atom, and I is the average bond length.[14]

As a result, $(MAMP)(MA)_3Pb_4I_{13}$ perovskite possesses a smaller average BAV and DI values (37.26 and 0.012) compared to $(BA)_2(MA)_3Pb_4I_{13}$ perovskite (60.18 and 0.022). The distortion of the inorganic slabs is greatly reduced compared to BA perovskites, which significantly decreased the antibonding hybridization, pushing up the valence band maximum and pushing down the conduction band minimum . As a result, MAMP quasi-2D perovskite possesses a smaller bandgap than BA perovskite.

Supplementary Note 2 | Details for SCAPS Simulation.

Different levels of energy disorder active layer will affect the carrier mobility, recombination rate, and ultimately bring different defect density (N_t). We selected four devices with different energy disorders to determine their corresponding N_t. The N_t value is: 3×10^{15} cm^{-3} (σ = 91 meV), 8×10^{15} cm^{-3} (σ = 98 meV), 5×10^{16} cm^{-3} (σ = 109 meV), and 1×10^{15} cm^{-3} (σ = 122 meV), respectively. The device performance for (MAMP)$MA_3Pb_4I_{13}$ perovskite with different energy disorder were simulated by SCAPS. The parameters of perovskite absorber layer were shown in the Supplementary Table 4.

Supplementary Methods

Characterization of perovskite single crystals and films

Ultraviolet photoelectron spectra of perovskite single crystals were obtained by Thermo Scientific ESCA Lab 250Xi, using Au as the standard sample for calibration. UPS measurements were carried out in an ultra-high-vacuum (UHV) chamber with a base pressure below 5×10^{-9} mbar. The photon line width was about 250 meV and the minimum spot size about 1 mm. He I (hv = 21.22 eV) emission source was employed and the helium pressure in the chamber during analysis was about 2×10^{-8} mbar. The work function (W_F) of perovskite single crystals can be calculated from following equation:

$$W_{\mathrm{F}} = hv - (E_{\mathrm{Ferni}} - E_{\mathrm{Cutoff}}) \quad (3)$$

where the E_{cutoff} is the steep edge position in the UPS spectrum. The valence band (E_V) and conduction band (E_C) of the single crystals can be calculated by the equation:

$$E_V = W_{\mathrm{F}} + VBM \quad (4)$$

$$E_C = W_{\mathrm{F}} + VBM - E_{\mathrm{g}} \quad (5)$$

where E_g is the optical bandgap extracted from the absorption spectra.

UV-Visible absorption spectra of perovskite single crystal were captured by measuring the diffuse reflectance spectra of perovskite single crystal using a Cary 5000 (Agilent Technologies). The absorption spectra of quasi-2D perovskite films were carried out on a Lambda 950 UV-vis spectrophotometer.

X-ray diffraction patterns of perovskite single crystals and films were measured by Rigaku_Dmax_2500 with a step of 0.02 °from 5 °to 50 °. The perovskites powders were obtained through gridding perovskites bulks by an agate pestle and mortar. The lines were Cu $K_{\alpha1}$ and Cu $K_{\alpha2}$.

Scanning electron microscopy. The cross-section images of perovskite solar cell device and surface morphology of perovskite films were characterized by field-

emission Scanning Electron Microscopy (SEM; JSM-7500F, JEOL). The energy dispersive spectroscopy (EDS) was conducted by using an EDS device connected to FESEM.

Photoluminescence (PL) spectra. The steady-state PL spectra were measured by fluorescence spectrophotometer (Edinburgh instruments FLS 980) provided by a xenon lamp, and perovskite films were excited at 475 nm. The excitation source was a laser diode (450 nm). The TRPL lifetimes are fitted by a double exponential function as follows,

$$f(t) = A_1 \times e^{\frac{-t}{\tau_1}} + A_2 \times e^{\frac{-t}{\tau_2}}. \quad (6)$$

And the average TRPL lifetimes is calculated by the equation,

$$\tau_{\text{ave}} = \frac{A_1{\tau_1}^2 + A_2{\tau_2}^2}{A_1\tau_1 + A_2\tau_2}. \quad (7)$$

The initially photogenerated carrier density (n_0) of perovskite films could be evaluated by the equation:

$$n_0 = \frac{E\lambda\alpha(\lambda)}{hcA(1-R_{\text{pump}})} \quad (8)$$

$$\alpha(\lambda) = -\frac{1}{l}\ln\frac{T}{1-R} \quad (9)$$

Where E was the energy contained in the excited pump pulse with a wave length of λ; $\alpha(\lambda)$ and R_{pump} represented the absorption coefficient and the reflectance of the excited pump pulse; A, T, R, and l was the effective area, transmittance, reflectivity and thickness of perovskite films, respectively.

In general, the recombination dynamics could be described by the following equation,

$$\frac{\mathrm{d}n_0(t)}{\mathrm{d}t} = -k_1 n - k_2 n^2 - k_3 n^3 \quad (10)$$

Where k_1, k_2 and k_3 represent the first, second and three order rate constants for trap-assisted, bimolecular recombination and Auger recombination, respectively. k_1, k_2 and k_3 were extracted by global fits of density dependent TRPL data by using the equation.

Relative dielectric constant measurement. Capacitance was determined using

impedance analyzer (Agilent4294A) over a wide frequency range from 100 Hz up to 60 MHz. The relative dielectric constant of the prepared samples was calculated using the formula,

$$\varepsilon = \frac{Cd}{\varepsilon_0 S}. \tag{11}$$

Where *C* is the measured capacitance, *d* is the thickness of the single crystals, *S* is the surface area, and ε_0 is the permittivity constant in free space.

The space-charge-limited current measurement. Trap density and mobility were measured by SCLC model, using an electron only device structure of ITO/TiO_2/perovskite/PCBM/Ag. The dark current density-voltage (*J*-*V*) characteristics of the electron-only devices were measured by a Keithley 2400 sourcemeter. The electron mobility then extracts from the child region with the equation of

$$I = \frac{9\varepsilon_0 \varepsilon_r \mu S V^2}{8L^3}. \tag{12}$$

Where *I* is the device current, ε_r is the relative dielectric constant, ε_0 is the vacuum permittivity, *S* is the electrode area, *L* is the film thickness.

A rapid nonlinear trap-filled limit (TFL) rise was observed, which represents all the available defect states were filled by the injected carriers. The onset voltage, V_{TFL}, was linearly proportional to the density of defect states,

$$N_t = \frac{2\varepsilon_0 \varepsilon_r V_{TFL}}{qL^2}. \tag{13}$$

Thermal admittance spectroscopy analysis was obtained by capacitance-frequency curve using PARSTAT 4000 was used to measure the capacitance-frequency curve. The energy profile of trap density of states could be fitted from the equation:

$$N_t(E_\omega) = -\frac{V_{bi}}{eW}\frac{dC}{d\omega}\frac{\omega}{k_B T} \tag{14}$$

Where V_{bi} is the built in voltage, ω is angular frequency, *e* is the elementary charge, *W* is the depletion width, *C* is the capacitance, k_B is the Boltzmann constant and *T* is the temperature.

Characterization of perovskite devices

The steady-state photocurrent curves were measured under simulated AM 1.5G irradiation (100 mW cm^{-2}) using a xenon-lamp-based solar simulator (Oriel 67005, 150 W Solar Simulator). For each measurement, the lamp was turned on and the testing started 30 min later. A Schott visible-color, glass-filtered (KG5 color-filtered) Si diode (Hamamatsu S1133) was used to calibrate the light intensity before the photocurrent measurement.

Impedance spectrum was measured by the electrochemical workstation. The light source was simulated by a Xenon-lamp-based solar simulator (Enli Tech), and the illumination intensity was tuned by filters. The impedance spectra was recorded by the E4980A Precision LCR Meter from Agilent with homemade software in the frequency range of 2000 to 0.2 kHz. For transport resistance (R_{ct}) and recombination resistance (R_{rec}) measurements, the devices ware applied with a bias voltage equaled to V_{oc} under dark conditions. For energetic disorder parameter extraction, the devices were measured under different light intensity, and the applied bias voltage equaled the V_{oc} under each light intensity.

Electroluminescence quantum efficiency measurements were conducted using a Newport QE measurement kit via a system combining a xenon lamp, a monochromator, a chopper and a lock-in amplifier together with a calibrated silicon photodetector. For light-emitting diodes device characterizations, a fiber integration sphere (FOIS-1-FL) coupled with a QE65 Pro spectrometer was used for the light output measurement. The devices were measured from zero bias to forward bias with a scanning rate of 0.1 V s^{-1} and a dwell time of 1 s.

Transient photocurrent and transient photovoltage measurement were measured on a home-made system. A white light bias is generated from an array of diodes (100 mW cm^{-2}) and red light pulse diodes (0.05 s square pulse width, 100 ns rise and fall time, 5 mW cm^{-2}) controlled by a fast solid–state switch were used as the perturbation

source. TPC was measured using 20 Ω external series resistance to operate the device in short-circuit. Similarly, TPV was measured using 1 MΩ external series resistance to operate the device in open-circuit. The voltage dynamics on the resistors were recorded on a digital oscilloscope (Tektronix MDO3032). The perturbation red light source was set to a suitably low level to the white diodes array with light intensity equivalent to 100 mW cm^{-2} of a standard solar simulator.

Supplementary References

1 Stoumpos, C. C. *et al.* Ruddlesden-Popper hybrid lead iodide perovskite 2D homologous semiconductors. *Chem. Mater.* **8**, 2852-2867 (2016)

2 Mao, L. *et al.* Hybrid Dion-Jacobson 2D lead iodide perovskites. *J. Am. Chem. Soc.* **140**, 3775-3783 (2018).

3 Garcia-Belmonte, G., Boix, P. P., Bisquert, J., Sessolo, M. & Bolink, H. J. Simultaneous determination of carrier lifetime and electron density-of-states in P_3HT: PCBM organic solar cells under illumination by impedance spectroscopy. *Solar Energy Mater., Solar Cells* **94**, 366-375 (2010).

4 Stranks, S. D. *et al.* Electron-hole diffusion lengths exceeding 1 micrometer in an organometal trihalide perovskite absorber. *Science* **342**, 341-344 (2013).

5 Ramli, N. F. *et al.* in *AIP Conference Proceedings.* AIP Publishing **1838**, 020006 (2017).

6 Noh, J. H., Im, S. H., Heo, J. H., Mandal, T. N. & Seok, S. I. Chemical management for colorful, efficient, and stable inorganic-organic hybrid nanostructured solar cells. *Nano lett.* **13**, 1764-1769 (2013).

7 Brivio, F., Walker, A. & Walsh, A. *Apl. Mater.* **1**, 042111 (2013).

8 Tsai, H. *et al.* High-efficiency two-dimensional Ruddlesden-Popper perovskite solar cells. *Nature* **536**, 312-316 (2016).

9 Kresse, G. & Furthmuller, J. Efficient iterative schemes for ab initio total-energy calculations using a plane-wave basis set. *Phys. Rev. B: Condens. Matter Mater. Phys.* **54**, 11169-11186 (1996).

10 Perdew, J. P., Burke, K. & Ernzerhof, M. Generalized gradient approximation made simple. *Phys. Rev. Lett.* **77**, 3865-3868 (1996).

11 Blochl, P. E. Projector augmented-wave method. *Phys. Rev. B: Condens. Matter Mater. Phys.* **50**, 17953-17979 (1994).

12 Grimme, S., Antony, J., Ehrlich, S. & Krieg, H. Consistent and accurate Ab initio parametrization of density functional dispersion correction (DFT-D) for the 94 elements H-Pu. *J. Chem. Phys.* **132**, 154104 (2010).

13 Robinson, K., Gibbs, G. V. & Ribbe, P. H. Quadratic elongation: a quantitative measure of distortion in coordination polyhedra. *Science* **172**, 567-570 (1971).

14 Baur, W. H. Geometry of polyhedral distortions. Predictive relationships for the phosphate group. *Acta Crystallogr. B* **30**, 1195-1215 (1974).
